# Spurious Absorption Frequency Appearance Due to Frequency Conversion Processes in Pulsed THz TDS Problems

**DOI:** 10.3390/s20071859

**Published:** 2020-03-27

**Authors:** Vyacheslav A. Trofimov, Nan-Nan Wang, Jing-Hui Qiu, Svetlana A. Varentsova

**Affiliations:** 1 Department of Microwave Engineering, Harbin Institute of Technology, Harbin 150001, China; wangnn@hit.edu.cn (N.-N.W.); qiujh@hit.edu.cn (J.-H.Q.); 2 Faculty of Computational Mathematics and Cybernetics, Lomonosov Moscow State University, Leninskiye Gory, Moscow 119992, Russia; svarentsova@gmail.com

**Keywords:** pulsed THz Time-Domain Spectroscopy, broadband THz pulse, THz CW radiation, frequency conversion, substance emission, frequency doubling, linear combination of the absorption frequencies, detection and identification of substance

## Abstract

The appearance of the spurious absorption frequencies caused by the frequency conversion process at the broadband THz pulse propagation in a medium is theoretically and experimentally discussed. The spurious absorption frequencies appear due to both the frequency doubling and generation of waves with sum or difference frequency. Such generation might occur because of the nonlinear response of a medium or its non-instantaneous response. This phenomenon is confirmed by the results of a few physical experiments provided with the THz CW signals and broadband THz pulses that are transmitted through the ordinary or dangerous substances. A high correlation between the time-dependent spectral intensities for the basic frequency and generated frequencies is demonstrated while using the computer simulation results. This feature of the frequency conversion might be used for the detection and identification of a substance.

## 1. Literature review and introduction

The THz time-domain spectroscopy (THz TDS) is a non-destructive tool that is very popular nowadays for both basic research and the security screening and for industrial applications also. Is well known that the THz pulsed TDS is traditionally used for the detection and identification of explosive and illicit drug [1,2,3,4,5,6,7,8,9,10]. Moreover, it is currently considered to be a new powerful tool for application in the chemical sciences and molecular structure investigations [11,12,13,14], nondestructive inspections [15,16,17,18], thickness measurements [19,20], art investigations [21,22], pharmaceutical and biomedical applications [23,24,25,26], and food quality control [27,28,29]. In [30], a comprehensive review of the history and state of the art of the GaN-based devices for THz applications is provided. One important limitation of the most THz sources and detectors should also be noted. Namely, they are restricted by their low conversion efficiency. Great efforts have therefore been made to improve the efficiency of THz sources and detectors [31,32]. 

As a rule, substance identification is carried out based on a comparison of the absorption frequencies of a substance under investigation with a set of known absorption frequencies from a database. We call this identification technique the standard THz TDS method. Obviously, this method has well-known disadvantages. For example, if the object is masked by some non-opaque covering or hidden under the packaging material, then the spectrum of the measured THz signal might be significantly distorted [33,34]. Moreover, such factors as inhomogeneity of the substance surface and atmospheric humidity (and many other factors) also significantly distort the spectra of the measured THz pulse [35,36,37,38]. Moreover, the spurious artifacts associated with the experimental setups and with TDS THz system as well as with signal processing and sample geometries, also bring difficulties at measuring of the optical properties of the substance under investigation [39,40,41,42]. In all of these cases, the substance identification by means of the standard THz TDS is ineffective.

In addition, some non-opaque packaging materials (paper, plastic material, clothes, etc.) usually cover an examined substance [43]. Often, these covers possess both density fluctuations and sample thickness variations in the sub-millimeter scale of length and, therefore, they can be considered to be a disordered photonic structure in the THz frequency range. Consequently, one can appear the false absorption frequencies, which are caused by inhomogeneities, in the spectrum of the broadband THz pulse transmitted through or reflected from these ordinary materials and these frequencies may coincide with those of the dangerous substances [44,45,46]. Thus, the appearance of the false absorption frequencies under the THz TDS is an ordinary phenomenon and recognizing of the true absorption frequencies inherent to a substance under consideration is an urgent problem. We have also proposed an efficient tool for overcoming of influence of most of these factors on the detection and identification of a substance. Our approach is based on an analysis of the time-dependent spectral intensities (SDA-method) on both the absorption frequencies and the emission ones. Earlier, the SDA-method was successfully applied for the detection and identification of neutral and dangerous substances under real conditions [47,48,49,50,51,52,53,54], including the cases of very noisy THz signal and a sample with inhomogeneous surface. Therefore, we also follow this approach below.

There are also other physical mechanisms, which are not associated with the factors mentioned above and they lead to observing of the spurious absorption frequencies in the spectrum of the pulse transmitted through or reflected from a substance. They may appear due to the frequency conversion processes at resonant or non-resonant interaction of the THz pulse with a substance or due to non-instantaneous response of a medium. One of them is the cascade mechanism of the high energy level excitation of molecules [55,56]. It leads to the generation of the frequencies not belonging to the incident pulse spectrum. Another one, which is considered below, is a frequency conversion, because of the nonlinear response of a medium. For example, the second harmonic generation (SHG), or generation of waves with the frequencies, which are a linear combination of the frequencies that belong to the incident pulse spectrum, may appear due to the quadratic nonlinear response of a medium. It is important to stress that the frequency conversion or interaction of the Fourier harmonics of a wave packet can simultaneously occur because the incident THz pulse possesses broadband spectrum and the pulse interaction with a medium is essentially non-instantaneous. Moreover, a part of them might be observed after the action of a THz pulse transmitted through/reflected from a medium because of the possible delay of the emission of a medium. This feature distinguishes the frequency conversion of the broadband THz pulse from processes occurring in nonlinear optics, where it occurs for monochromatic optical wave, or from frequency changing when the monochromatic wave interacts with two-level medium, for example. 

It should be stressed that the spurious absorption frequencies that appear under the frequencies up-conversion process (for example, at SHG) may be used as a new effective tool for the detection and identification of the substance. We illustrated such a possibility in [57,58], where a description of the nonlinear response of a medium was provided while using density matrix formalism [59] and the THz pulse propagation is described by one-dimensional (1D) Maxwell’s equations. A single substance layer was placed between two coverings, which consisted of linear layers with random dielectric permittivity. The appearance of the doubled absorption frequency under non-stationary interaction of the THz broadband pulse with multi-levels molecules was demonstrated in both transmission and reflection mode. Analyzing time-dependent spectral intensity at the doubled frequency, we showed a possibility for the substance detection and identification despite covering with random parameters. 

In this paper, we demonstrate the appearance of the spurious absorption frequencies under the frequency conversion process that is caused by nonlinear (quadratic) response of a medium. In contrast to the previous papers, in the theoretical analysis, we consider the instantaneous response of a medium under the action of different types of signals consisting of several sub-pulses with narrow and broad spectra. Such a signal is a typical one for the pulsed THz TDS of the substance made similar to a tablet. A high correlation between the time-dependent spectral intensities for the basic frequency and generated frequencies (doubled frequency, or sum frequency, or difference frequency) is demonstrated for various types of the signals. This is the basis for using spurious absorption frequencies or the emission frequencies as the frequencies for the detection and identification of a substance.

We discuss the results of a few physical experiments carried out with broadband THz pulses and narrowband CW radiation, transmitted through the ordinary materials, to verify our theoretical conclusions about the appearance of the spurious absorption frequencies caused by the frequency conversion processes due to the nonlinear or non-stationary process and to demonstrate their appearance (soap, paper, chocolate, and plastic). It should be stressed that the experiments with ordinary materials were carried out in the room atmosphere with relative humidity of about 30%. It means that the measured THz signals are noisy, and this brings our investigation close to reality. Using the standard THz TDS method under such a condition can lead to a large number of false positive alarms during security screening, which renders its effectiveness low. At the same time, a possibility of the substance detection and identification by means of SDA-method together with several integral correlation criteria (ICC) using noisy THz signals with SNR about 0.5 was demonstrated in [47,48,51]. Additionally, we use this technique in the current paper.

The measurements for narrowband THz CW radiation transmitted through the samples with aspirin, soap, paper, and chocolate were provided in the University of Dayton (Dayton, OH, USA). The measurements for broadband THz pulse transmitted through the polyethylene and paper were made in the Military University of Technology (Warsaw, Poland) and in the South China Normal University (Guangzhou, China), respectively. The measurements for the broadband THz pulse interaction with 2,4-DNT were made at the Capital Normal University (Beijing, China). The physical experiments with transmission of the broadband THz pulses through the soap and chocolate were measured in the Institute of Spectroscopy RAS (Moscow, Troitsk, Russian).

We use the results of several physical experiments performed at different times and in different labs to illustrate that the conclusions do not depend on particularities of the THz setup. We stress that for the measurements of the broadband THz pulse the similar setups in various labs were used. We specify their types and characteristics in Section 3. 

## 2. Theoretical Analysis

**Figure 1:**
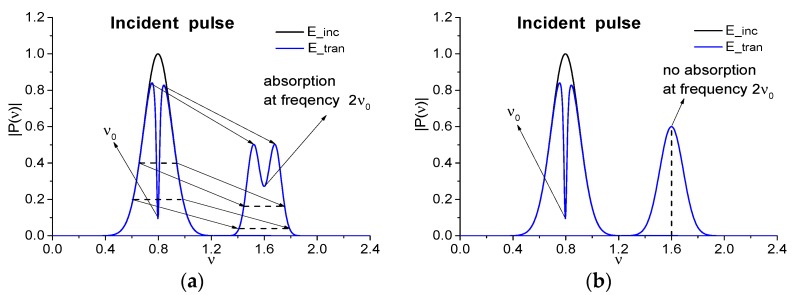
Scheme of verification of the substance absorption frequencies. The absorption frequencies $\nu_{0}$, $2\nu_{0}$ occurring in the spectrum of the incident broadband pulse, transmitted through a substance, are seen in the spectrum of the incident narrowband signal (E_tran), transmitted through this substance, at changing its carrier frequency (**a**). Thus, the frequency $2\nu_{0}$ is a true absorption frequency. (**b**) demonstrates that the frequency $2\nu_{0}$ is induced by the up-conversion process (the spurious frequency), because it is not observed in the spectrum of the incident narrowband signal transmitted through a substance. Black line denotes the spectrum of the incident narrowband signal (E_inc).

As we mentioned in the Introduction, the process of a broadband THz pulse interaction with a substance because of both non-instantaneous response of a medium, and its nonlinear response, as well as its resonance response, can lead, in particular, to the appearance of both the spurious absorption frequencies and the substance emission frequencies in the spectrum of the pulse transmitted through a medium. Let us remind that frequency conversion efficiency depends on a spectral brightness of the corresponding Fourier harmonics $\nu_{0}$. Therefore, if we consider a process of the frequency doubling under the propagation of the broadband pulse and the frequency is the absorption one of a substance, then the spectrum of the pulse, which is transmitted through a medium, contains a minimum near the frequency $2\nu_{0}$ (see Figure 1a, an accordance between the intensities is schematically depicted by arrows). Thus, we will observe the induced absorption frequency, if the spectral resolution of the measurement device is high enough.

In Figure 1 the scheme of verification of the substance absorption frequencies $\nu_{0}$ (the basic frequency) and $2\nu_{0}$ (the doubled frequency) (whether it is true or false), as observed in the spectrum of the incident broadband pulse, transmitted through the substance, is shown. With this aim, the narrowband pulse should be used (black line in Figure 1a). While shifting its current frequency along the frequency axis, one can observe the presence or absence of the pulse energy absorption at the frequency $2\nu_{0}$. If the pulse energy absorption is present at the frequency (Figure 1a, $\nu_{0}$, $2\nu_{0}$), then it means that we observe the true absorption frequency of a substance. If there is no minimum in the spectrum at the doubled frequency $2\nu_{0}$ at using the probing narrowband THz CW radiation (Figure 1b), then the absorption frequency $2\nu_{0}$ occurring in Figure 1a at the broadband pulse transmission through a medium is induced by the frequency doubling process. Therefore, this frequency is the spurious one.

Before the discussion of results of the theoretical analysis of an appearance of the spurious absorption (or, may be, emission) frequencies we make two remarks. Firstly, for definitely, below we consider a transmission of the incident pulse through a medium with the quadratic nonlinear response in the approximation of the so-called optical thin layer. In this case, at the supposing of the instantaneous response of a medium, we can write the following expression:

$$E(t)=E_{inc}(t)+\chi^{\left(2\right)}E_{inc}^{2}(t)$$

for the electric field strength of the pulse transmitted through a medium. A dimensionless parameter $\chi^{\left(2\right)}$ characterizes the quadratic susceptibility of a medium. To illustrate the appearing of the basic frequency conversion, we choose this parameter equal to unity. However, in practice, this parameter can be significantly less than unity; its value depends on both the properties of a medium and the incident pulse maximal intensity. 

Secondly, we consider two types of the artificial signal while taking into account the structure of the real THz pulse transmitted through the layer of a medium. Depending on its thickness, this pulse can contain separate or overlapping sub-pulses.

### 2.1. Frequency Conversion Near the Spectral Maximum of Artificial Signals

Let us consider the artificial incident pulse $E_{inc}(t)$ that is the sum of four terms and has the following structure:

(1) $$\begin{array}{l} E_{inc}(t)=A_{k1}\cdot \exp\left(-{\left(\frac{\left(t-t_{k1}\right)}{\tau_{k1}}\right)}^{2}\right)\cdot \cos(2\pi \nu_{1}t)+A_{k2}\cdot \exp\left(-{\left(\frac{\left(t-t_{k2}\right)}{\tau_{k2}}\right)}^{2}\right)\cdot \cos(2\pi \nu_{2}t)+\\ +A_{k3}\cdot \exp\left(-{\left(\frac{\left(t-t_{k3}\right)}{\tau_{k3}}\right)}^{2}\right)\cdot \cos(2\pi \nu_{3}t)+A_{k4}\cdot \exp\left(-{\left(\frac{\left(t-t_{k4}\right)}{\tau_{k4}}\right)}^{2}\right)\cdot \cos(2\pi \nu_{4}t),k=1,2. \end{array}$$

Here, $A_{ki}$ is the amplitude of the corresponding sub-pulse, $t_{ki}$ is its center, and $\tau_{ki}$ is the half-duration of the sub-pulse, where $i=\bar{1,4}$. The carrier frequencies $\nu_{k}$ are fixed for all artificial signals and have the values: $\nu_{1}=0.8$, $\nu_{2}=1.2$, $\nu_{3}=1.4$, and $\nu_{4}=1.8$. We will call them as the basic frequencies. In Table 1, the parameters of two artificial signals $E_{inc}(t)$ are shown. Let us note that a time t and the frequency ν and $A_{ki}$ are dimensionless variables.

**Figure 2:**
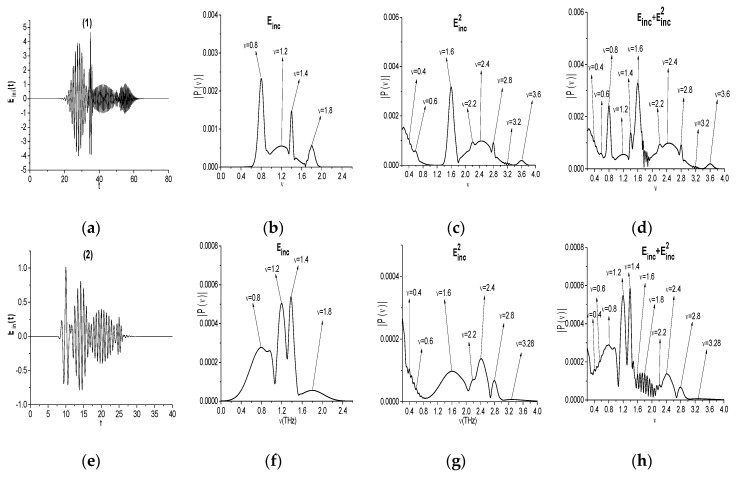
Artificial pulses $E_{inc}(t)$ (**a**,**e**), their spectra (**b**,**f**), and the signal ${E_{inc}}^{2}(t)$ spectra (**c**,**g**) and the signal $E_{inc}(t)+{E_{inc}}^{2}(t)$ spectra (**d**,**h**).

The pulse $E_{inc}(t)$, its spectrum, and the spectra of the signals ${E_{inc}}^{2}(t)$ and $E_{inc}(t)+{E_{inc}}^{2}(t)$ are shown in Figure 2 for different values of parameters $A_{ki}$, $t_{ki}$, $\tau_{ki}$, as depicted in Table 1. 

It is easy to see that the artificial signal $E_{inc}(t)$ (a) consists of four intersecting sub-pulses with three narrow spectra and one broad spectrum with the pulse duration $\tau_{12}=2$. The sub-pulses spectra also partially overlap. Nevertheless, the signal $E_{inc}(t)$ spectrum (b) possesses four pronounced maxima at the basic frequencies and small maximum near the frequency 1.5. In the signal ${E_{inc}}^{2}(t)$ spectrum (c) there are maxima at the doubled basic frequencies *ν* = 1.6, 2.4, 2.8, 3.6, due to the quadratic nonlinear response of a medium. Moreover, one can also see the spectral maxima at the frequencies *ν* = 0.4, 0.6, 2.2, 3.2, which are the linear combinations of the basic frequencies (b). An appearance of these frequencies in the spectrum is discussed below. In (c), we can also observe the broadening of the incident pulse spectrum in the high (*ν* > 2.0) and low (*ν* < 0.6) frequency ranges. 

The second artificial signal $E_{inc}(t)$ (e) also consists of intersecting sub-pulses, but their spectra essentially overlap that results in appearance of the additional spectral maximum at the frequency *ν* = 0.96 (b1). The signal ${E_{inc}}^{2}(t)$ spectrum (g) obviously contains maxima at the doubled frequencies *ν* = 1.6, 2.4, 2.8. At the same time, in (g), the maximum at the doubled frequency *ν* = 3.6 is absent, and one can see additional spectral maximum at the frequency *ν* = 3.28. This is a result of a competition between processes of SHG (*ν* = 3.6 = 1.8·2) and sum frequency wave generation (SFG) $\nu_{sum}$ = 3.2 = 1.8 + 1.4. The additional spectral maxima can be also observed as a result of the difference frequency wave generation (DFG): $\nu_{dif}$ = 0.4 = 1.2 − 0.8, 0.6 = 1.8 − 1.2, 2.2 (b1). However, some of them can be obtained due to the generation of waves with half basic frequencies. For example, the frequency *ν* = 0.4 might occur as a generation of a wave with the frequency *ν* = 0.8/2. (Figure 2(g)). We will denote these frequencies as $\nu_{sum}$, $\nu_{dif}$, respectively, and $\nu_{prod}$—as a linear combination of the basic frequencies. Thus, various processes of wave generation give contribution to the appearance of a wave at certain frequency.

Obviously, the spectral maxima of the signal $E_{inc}(t)+{E_{inc}}^{2}(t)$ (Figure 2(d,h) coincide with the maxima of the corresponding signals $E_{inc}(t)$ (b,b1) and ${E_{inc}}^{2}(t)$ (c,g). Therefore, in the following Sections, we will analyze the evolution of the time-dependent spectral amplitudes (spectral line dynamics) at the basic and doubled frequencies as well as at the additional emission frequencies for the signals $E_{inc}(t)$ and ${E_{inc}}^{2}(t)$. The correlation between these spectral intensities is of most interest for us.

#### 2.1.1. Correlation between the Spectral Line Dynamics at the Basic and Doubled Frequencies for the Signal Consisting of Sub-Pulses with Three Narrow Spectra and One Broad Spectrum (Example 1)

**Figure 3:**
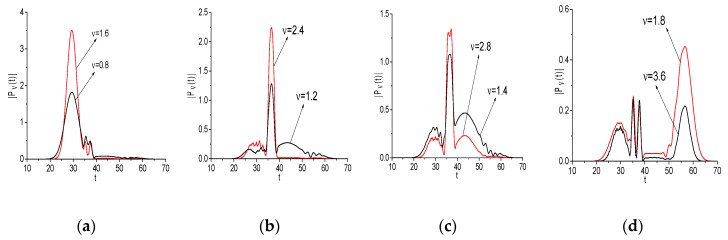
Evolution of the spectral amplitude modulus at the basic frequency $\nu_{base}$ = 0.8, 1.2, 1.4, 1.8 (black line, signal $E_{inc}(t)$) and doubled frequency *ν* = 1.6, 2.4, 2.8, 3.6 (red line, signal ${E_{inc}}^{2}(t)$), as depicted in (**a**–**d**), respectively.

In this Section, we investigate the evolution of the spectral amplitudes—square root from spectral intensities—at the basic, doubled, and other emission frequencies for the signals $E_{inc}(t)$ and ${E_{inc}}^{2}(t)$ (Example 1). The main aim of this investigation is a demonstration of high similarity between the spectral dynamics at these frequencies. As an example, the modulus of such spectral amplitude $\left|P_{\nu }(t)\right|$, $\left|P_{2\nu }(t)\right|$ evolution (their computation is described in Appendix A and in [44,45,46]) at the frequencies *ν* = (0.8, 1.6) (a), (1.2, 2.4) (b), (1.4, 1.8) (c), (1.8, 3.6) (d) is depicted in Figure 3. A high degree of similarity between time-dependent spectral amplitudes is observed in all cases (a–d), and we may expect their high correlation. Indeed, the corresponding correlation coefficient between them are close to unity: $c_{\nu ,2\nu }$ = 0.969 (a), 0.915 (b), 0.904 (c), 0.889 (d). Here we use the well-known expression:

(2) $$c_{\nu_{1},\nu_{2}}=\frac{\sum_{m=0}^{M-1}\left(|P_{\nu_{1}}(t_{m})-\bar{P_{\nu_{1}}}|)\cdot (|P_{\nu_{2}}(t_{m})-\bar{P_{\nu_{2}}}|\right)}{\left||P_{\nu_{1}}-\bar{P_{\nu_{1}}}||\cdot ||P_{\nu_{2}}-\bar{P_{\nu_{2}}}|\right|},\bar{P_{\nu_{1}}}=\sum_{m=0}^{M-1}\left|P_{\nu_{1}}(t_{m})\right|/M,\bar{P_{\nu_{2}}}=\sum_{m=0}^{M-1}\left|P_{\nu_{2}}(t_{m})\right|/M$$

where $\bar{P_{\nu_{1}}}$, $\bar{P_{\nu_{2}}}$ are the non-zero mean values of the spectral amplitudes $\left|P_{\nu_{1}}\right|$ and $\left|P_{\nu_{2}}\right|$. Subtracting of these values from the spectral dynamics in expression (2) is necessary to eliminate their influence on the correlation coefficient $c_{\nu_{1},\nu_{2}}$. Here M denotes the number of time moments in the spectral dynamics $\left|P_{\nu_{1}}\right|$, $\left|P_{\nu_{2}}\right|$, which depends on the dynamics construction parameters—the window length T and its shift Δ along the signal under investigation. With accordance to our previous articles ([44,45,46]), these parameters are chosen as T = 2.8 and Δ = 0.2 dimensionless units.

**Figure 4:**
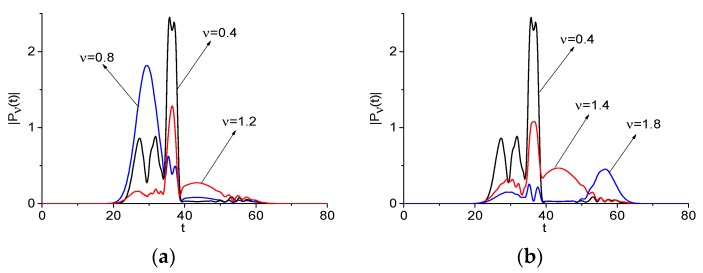
Evolution of the spectral amplitude modulus at the difference frequency $\nu_{dif}$ = 0.4 (black line, signal $E_{inc}^{2}(t)$) and basic one $\nu_{base}$ = (0.8, 1.2) (**a**), and (1.8, 1.4) (**b**) (blue and red lines, signal $E_{inc}(t)$), respectively.

The DFG may be obtained by two pairs of Fourier harmonics interaction: between the harmonics with frequencies *ν* = 1.2 and *ν* = 0.8; as well as *ν* = 1.8 and *ν* = 1.4, respectively: $\nu_{dif}$ = 0.4 = 1.2 − 0.8 = 1.8 − 1.4. Each of these pairs might give a contribution to the frequency $\nu_{dif}$ = 0.4 generation. To clarify which contribution occurs and in which time interval, we depict the spectral dynamics at the emission frequency *ν* = 0.4 together with the spectral dynamics at the basic frequencies *ν* = 0.8, 1.2, and *ν* = 1.4, 1.8 in Figure 4a,b, respectively. Table 2 provides the corresponding correlation coefficients $c_{\nu_{base},\nu_{dif}}$ (Example 1). The maximal correlation occurs between the DFG with $\nu_{dif}$ = 0.4 and the spectral line dynamics computed at the basic frequencies: $c_{1.2,0.4}$ = 0.884, $c_{1.4,0.4}$ = 0.796. 

When comparing the spectral line dynamics, one can see that the contribution of various basic frequencies to generation process changes in different time intervals. Accordingly, one can see in Figure 4 that the generation of the DFG with $\nu_{dif}$ = 0.4 due to the interaction of the harmonics at the basic frequencies *ν* = 0.8, 1.2 (a) and *ν* = 1.4, 1.8 (b) is clearly pronounced in the time interval t = [20, 40]. This is confirmed by the corresponding correlation coefficients: they increase in this time interval up to the values $c_{1.2,0.4}$ = 0.953 (Figure 4a), $c_{1.4,0.4}$ = 0.956 (Figure 4b). At the same time, in the short time interval t = [20, 34], the generation of the frequency $\nu_{dif}$ = 0.4 is defined by the intensity evolution at the frequency *ν* = 1.2 ($c_{1.2,0.4}$ = 0.958), in the time interval t = [34, 40]—by that at the frequency *ν* = 0.8 ($c_{0.8,0.4}$ = 0.95) (Figure 4a). In Figure 4b, the short time interval t = [20, 34] the generation of the frequency $\nu_{dif}$ = 0.4 is defined by the intensity evolution at the frequency *ν* = 1.8 ($c_{1.8,0.4}$ = 0.93), in the time interval t = [34, 40]—by that at the frequency *ν* = 1.4 ($c_{1.4,0.4}$ = 0.965). In the time interval t = [40, 70], the correlation coefficients decrease: $c_{1.2,0.4}$ = 0.712 (a), $c_{1.4,0.4}$ = 0.648 (b). Note that, in the short time interval t ≈ [50, 65] (Figure 4b), the generation of the frequency $\nu_{dif}$= 0.4 is defined by the intensity evolution at the frequency *ν* = 1.4. 

**Figure 5:**
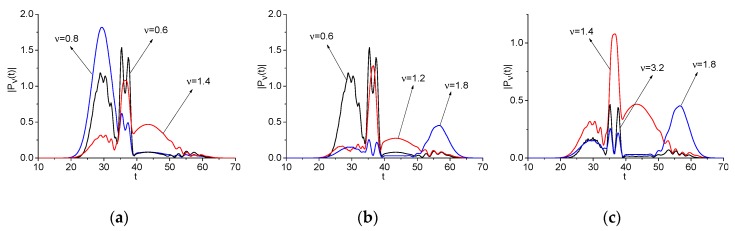
Evolution of the spectral amplitude modulus at the difference frequency $\nu_{dif}$ = 0.6 (black line (**a**), (**b**), signal $E_{inc}^{2}(t)$) and the sum frequency $\nu_{sum}$ = 3.2 (black line (**c**), signal $E_{inc}^{2}(t)$) and basic one $\nu_{base}$ = (1.4, 0.8) (**a**), and (1.2, 1.8) (**b**), and $\nu_{base}$ = (1.4, 1.8) (red and blue lines (**c**), signal $E_{inc}^{2}(t)$), respectively.

For further confirmation, we consider the DFG with frequency $\nu_{dif}$ = 0.6, which is the linear combinations of three generation processes: (i) frequency generation via interaction of two basic (carrier) frequencies $\nu_{base}$ = 1.4, 0.8; (ii) similar process with other pair of carrier frequencies $\nu_{base}$ = 1.8, 1.2; and, (iii) generation of wave with half frequency from the basic one $\nu_{base}$ = 0.6 = 1.2/2. Each of these processes may contribute to the frequency $\nu_{dif}$ = 0.6 generation process. Additionally, we consider the SFG $\nu_{sum}$ = 3.2 = 1.8 + 1.4. Figure 5 depicts the evolution of modulus of the spectral amplitudes of all frequencies *ν* = 0.6. We see the same features of the generation processes as in the previous case depicted in Figure 4. Therefore, different frequencies play determining role in various time intervals. The maximal correlation coefficients are for the spectral line dynamics at the basic frequency $\nu_{base}$ = 0.8 (a), 1.2 (b) and the difference frequency $\nu_{dif}$ = 0.6. They are equal to $c_{0.8,0.6}$ = 0.839, $c_{1.2,0.6}$ = 0.688 (Table 2, Example 1), respectively. Thus, the half frequency generation from the basic one $\nu_{base}$ = 1.2 can also contribute to the frequency $\nu_{dif}$ = 0.6 generation process.

In Figure 5c, we see a high correlation between the spectral line dynamic at the basic frequency $\nu_{base}$ = 1.8 and the sum frequency $\nu_{sum}$ = 3.2 if a time is less than 40 units. However, if a time is greater than 50 units, then an evolution of the spectral line dynamics at the basic frequency $\nu_{base}$ = 1.4 defines a process of the sum frequency generation. However, in the time interval t = [35, 40] units, the sum frequency generation is defined by the spectral intensity evolution at the basic frequency $\nu_{base}$ = 1.8. Nevertheless, the correlation coefficient between the spectral line dynamics at the basic frequency $\nu_{base}$ = 1.4 and the sum frequency $\nu_{sum}$ = 3.2 is equal to $c_{1.4,3.2}$ = 0.66. 

#### 2.1.2. Correlation between the Spectral Line Dynamics at the Basic and Doubled Frequencies for the Signal Consisting of Sub-Pulses with Three Broad Spectra and One Narrow Spectrum (Example 2)

This Section is similar to the previous one. However, a principal distinction of the signal under consideration from previous one lies in the structure of the pulse: it consists of three broadband sub-pulses and a sub-pulse with narrow spectrum (Example 2, Table 2). Therefore, the pulse spectrum differs markedly from the spectrum of the pulse investigated in Section 2.1.1 (see Figure 2 (e,g)). One can see that the spectral intensity of the signal $E_{inc}^{2}(t)$ spectrum insignificantly changes in the frequency range *ν* = [3.0, 3.8] units. This is a consequence of the spectrum overlapping of two sub-pulses. In this case, we do not see a pronounced maximum at the doubled frequency *ν* = 3.6 in Figure 2 (g). Instead, the maximal spectral intensity is seen at the frequency *ν* = 3.28.

**Figure 6:**
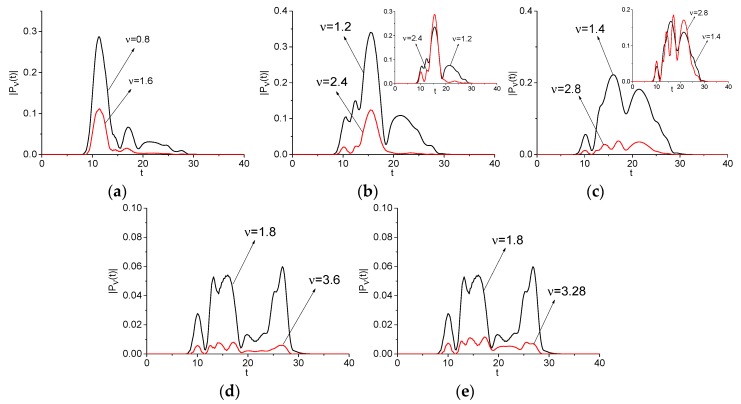
Evolution of the spectral amplitude modulus at the basic frequencies $\nu_{base}$ = 0.8, 1.2, 1.4, 1.8 (black line, signal $E_{inc}(t)$), and doubled ones *ν* = 1.6, 2.4, 2.8, 3.6 and frequency *ν* = 3.28 (**e**) (red lines, signal ${E_{inc}}^{2}(t)$, depicted in (**a**–**d**), respectively.

In Figure 6, the evolution of the spectral amplitude at the basic and doubled frequencies, and at the shifted frequency *ν* = 3.28 (e) is shown. One can see that an appearance of the wave with doubled frequency is delayed with respect to basic wave in any case and its trailing edge vanishes early than the spectral intensity at the basic frequency. This fact is in good agreement with the theory of SHG. 

Computed correlation coefficient between the corresponding spectral line dynamics demonstrates very high value: $c_{\nu ,2\nu }$ = 0.978 (a), 0.917 (b), 0.946 (c), 0.884 (d). It should be stressed that, for the case (e), we obtain the following correlation coefficient value $c_{1.8,3.28}$ = 0.881 and this value is very close to $c_{1.8,3.6}$ = 0.884. In our opinion, it means that two sub-pulse with overlapping spectra give their contributions to the process of SHG at the basic frequency *ν* = 1.8 and there is a generation of frequency band simultaneously. 

Note that the spectral amplitudes modulus $P_{\nu }\left(t\right)$, $P_{2\nu }\left(t\right)$ are computed according Equations (A1) and (A2) from Appendix A and are presented in Figure 6 in the non-normalized form. Therefore, it is not evident why the correlation coefficient for the spectral line dynamics depicted in (c–e) is so high. To clarify this fact, in Figure 6b,c, the corresponding spectral line dynamics are depicted in the additional panels that are normalized in L2-norm for convenience. In these additional panels, the high similarity in the shapes of the normalized spectral dynamics is evident and, therefore, the high values of the corresponding correlation coefficients are true. The same result takes place for the spectral line dynamics in (d), (e). 

**Figure 7:**
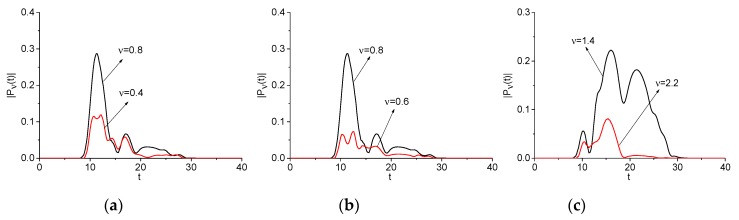
Evolution of the spectral amplitude modulus at the basic frequencies $\nu_{base}$ = 0.8 (**a**), (**b**), 1.4 (**c**) (black line, signal $E_{inc}(t)$) and difference frequencies $\nu_{dif}$ = 0.4 (**a**), 0.6 (**b**) and sum frequency $\nu_{sum}$ = 2.2 (**c**) (red line, signal ${E_{inc}}^{2}(t)$), respectively.

Figure 7 shows the spectral line dynamics at the difference frequency $\nu_{dif}$ = 0.4 = 1.2−0.8 = 1.8−1.4 (a), $\nu_{dif}$ = 0.6 = 1.4−0.8 = 1.8−1.2 (b), and sum frequency $\nu_{sum}$ = 2.2 = 0.8 + 1.4 (c), and basic frequencies *ν* = 0.8, 1.4, because these frequencies demonstrate the maximal correlation coefficients. All of their values are shown in Table 2 (Example 2). 

We see a high correlation between time-dependent spectral intensities at chosen frequencies: $c_{0.8,0.4}$ = 0.955 (a), $c_{0.8,0.6}$ = 0.886 (b), and $c_{1.4,2.2}$ = 0.718 (c). In Figure 7, we clearly see that these carrier frequencies give the main contribution to the generation process during time interval t ≈ [10, 20] units. Outside of this interval, the frequency generation occurs due other frequency mentioned above. Similar results were obtained for the artificial signal $E_{inc}(t)$, which consists of four narrow sub-pulses with non-overlapping spectra. Due to the simplicity of this case, no figures are given.

### 2.2. Frequency Conversion Near Minimum of the Pulse Spectrum 

Below, we investigate the frequency conversion if the frequency lies near the spectrum minimum (Examples 1 and 2 in Table 2). 

#### 2.2.1. The Artificial Signals $E_{inc}(t)$ Consisting from the Sub-Pulses (Examples 1 and 2)

We see in the artificial signal $E_{inc}(t)$ spectra, as depicted in Figure 2 (b,f), several minima at the basic frequencies $\nu_{base}$ = 0.96, 1.345, 1.46, 1.675 (b), 1.06, 1.31, and 1.53 (f). These minima are a consequence of the pulse structure: the pulse under consideration consists from the four sub-pulses. At the same time, in the signals ${E_{inc}}^{2}(t)$ and $E_{inc}(t)+{E_{inc}}^{2}(t)$ spectra (see Figure 2) one can observe the minima at the frequencies close to the corresponding doubled frequencies: *ν* = 1.8, 2.74, 2.86, 3.34 (c), (d), and 2.1, 2.67, 3.05 (g), (h). We see that, in some cases, the difference between values for doubled frequencies and observed frequencies might be significant. This is a result of the pulse structure: it consists of sub-pulses.

**Figure 8:**
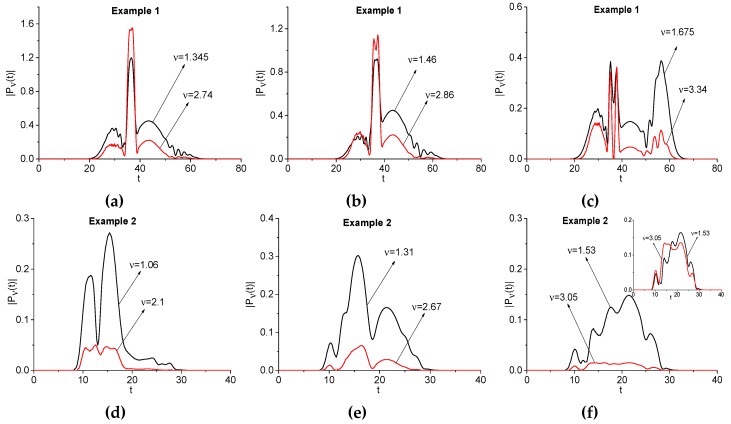
Evolution of the spectral amplitude modulus at the basic frequencies $\nu_{base}$ = 1.345, 1.46, 1.675 (Example 1) and $\nu_{base}$ = 1.06, 1.31, 1.53 (Example 2) (black lines, signal $E_{inc}(t)$) and at the frequencies close to doubled basic frequencies *ν* = 2.74, 2.86, 3.34 (Example 1) and *ν* = 2.1, 2.67, 3.05 (Example 2) (red lines, signal ${E_{inc}}^{2}(t)$), respectively depicted in (**a**–**f**).

The corresponding spectral line dynamics are shown in Figure 8 for the signals $E_{inc}(t)$ and ${E_{inc}}^{2}(t)$ computed for the Example 1 (a)–(c) and Example 2(d–f). We see high similarity between these spectral line dynamics. This fact is confirmed by the correlation coefficients, which are shown in Table 3. In most cases, they are very high: $c_{\nu ,2\nu }$ ≥ 0.9 (a), (b), (d)–(f). Maximal correlation coefficient occurs for time-dependent spectral intensities (e), (f): $c_{\nu ,2\nu }$ = 0.94 (e), 0.97 (f). As above (see Figure 6), the inset in (f) shows the same spectral line dynamics normalized in L2-norm, to clarify the high values of the corresponding correlation coefficients in (d–f). The additional panel in (f) confirms the high similarity in the shapes of the normalized spectral dynamics and, therefore, the high values of the corresponding correlation coefficients. The same result is valid for the spectral line dynamics in (d), (e). The dip in the spectral line dynamics that was computed at the doubled frequency *ν* = 2.86 and depicted in Figure 8b was caused by the pulse structure: the presence of the sub-pulses. 

The same results occur for the signal $E_{inc}(t)+{E_{inc}}^{2}(t)$ The spectral amplitude evolution computed for the doubled frequency is very similar to those computed for the basic ones. Thus, they (as well as the emission frequencies) can be used for the detection and identification of the substances, because the THz radiation at the frequencies belonging to the high-frequency range can propagate through the disordered structure with minimal distortion. 

#### 2.2.2. Artificial Signal without Sub-Pulse Structure (Example 3)

**Figure 9:**
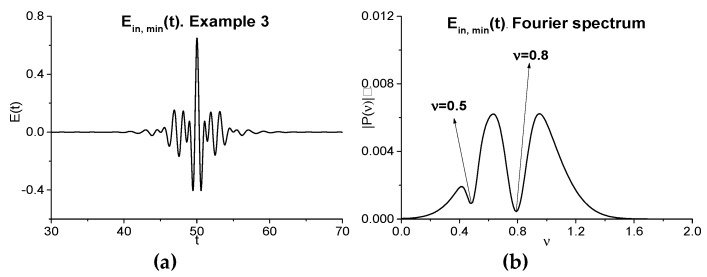

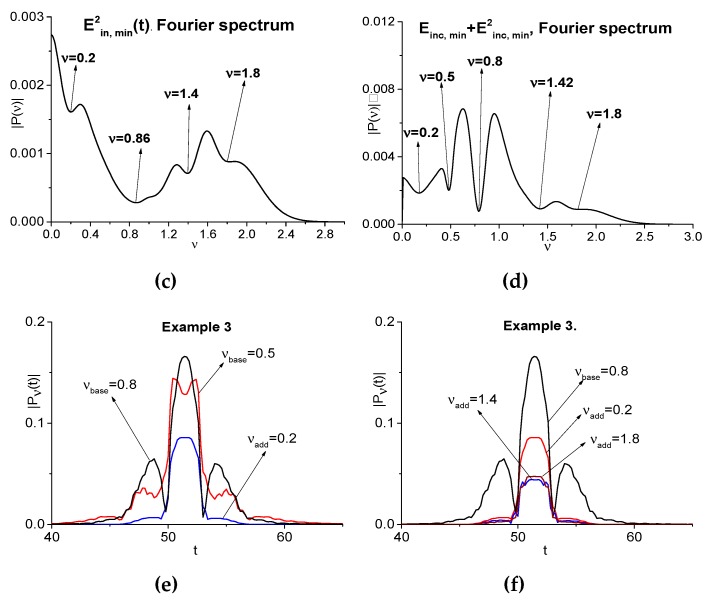
Artificial pulse $E_{inc,\min}(t)$ (**a**) and it spectrum, and the signal $E_{inc,\min}^{2}(t)$ spectrum (**c**), and the signal $E_{inc,\min}(t)+E_{inc,\min}^{2}(t)$ spectrum (**d**); spectral line dynamics at the basic frequencies $\nu_{base}$ = 0.5, 0.8 and additional frequencies *ν* = 0.2 (**e**), 0.2, 1.4, 1.8 (**f**).

In this Section, we consider the artificial signal $E_{inc,\min}(t)$ (Example 3), as depicted in Figure 9a, which does not possess the sub-pulse structure and contains two minima in its spectrum at the frequencies $\nu_{base}$ = 0.5, 0.8 (Figure 9b). Let us notice that such a structure of signal is present as a rule if we only consider the main THz pulse reflected from or transmitted through a medium. Below, we briefly describe a way of obtaining such a signal. For this aim, we consider the Gaussian pulse:

(3) $$E_{base}(t)=A_{b}\cdot \exp\left(-{\left(\frac{\left(t-t_{b}\right)}{\tau_{b}}\right)}^{2}\right)\cdot \cos(2\pi \nu_{b}t),0\leq t\leq 100$$

with $A_{b}=1$, $t_{b}=50$, $\tau_{b}=1$, $\nu_{b}=0.8$. Subsequently, we discretize function (3) on the time interval t = [0, 100] with the time step $h_{t}$, make the discrete Fourier transform and subtract from the sine- and cosine-Fourier transforms of the discrete function $E_{base}(t_{i}),i=1,\ldots ,N_{base}$, $N_{base}=100/h_{t}+1$ the discrete functions $G_{k}(\nu_{l})$, where:

(4) $$G_{k}(\nu )=B_{k}\cdot \exp\left(-{\left(\frac{\left(\nu -\nu_{k}\right)}{\Delta \nu_{k}}\right)}^{2}\right)\cdot \max|P_{b}|,k=1,2.$$

Here, the parameter $\max|P_{b}|$ denotes the maximal modulus of the spectral amplitude of the pulse $E_{base}(t)$ spectrum at the frequency $\nu_{k}$. The parameter $B_{k}$ describes the depth of the minimum at the frequency $\nu_{k}$ and it is equal to the following values: $B_{1}$ = 0.95, $B_{2}$ = 0.3. The parameter $\Delta \nu_{k}$ characterizes the absorption line spectral width and is equal to $\Delta \nu_{1}$ = 0.1, $\Delta \nu_{2}$ = 0.05. After the inverse Fourier transform, we obtain the pulse, being denoted as $E_{inc,\min}(t)$, with the spectral minima at the given frequencies $\nu_{1}=0.5$ and $\nu_{2}=0.8$. Additionally, in the signal $E_{inc,\min}(t)$ spectrum that is depicted in Figure 9b, one can see three maxima at the frequencies *ν* = 0.41, 0.63, 0.95. 

The signals $E_{inc,\min}^{2}(t)$ and $E_{inc,\min}(t)+{E_{inc,\min}}^{2}(t)$ spectra are shown in (c), (d). They possess several minima at the frequencies *ν* = 0.2 = 0.5·2 − 0.8, *ν* = 0.86, *ν* = 1.4 = 0.5·2 + 0.8/2, *ν* = 1.8 = 0.5·2+0.8 (c), and *ν* = 0.2, 0.5, 0.8, 1.42, 1.8 (c), part of which are the linear combinations of the basic frequencies. In each of the spectra, an emission at the doubled frequency *ν* = 0.5·2 is present.

The spectral line dynamics computed at the basic frequencies that correspond to minimal spectrum intensities and at the frequencies, which are produced by interaction of these spectral harmonics, are depicted in Figure 9e,f. We see that the main contribution gives the spectral harmonic at the frequency *ν* = 0.8. The corresponding correlation coefficients are shown in Table 4. All of them are high and greater than 0.9. 

Thus, we see that the spurious absorption frequencies that belong to the spectrum of the transmitted broadband pulse, which initially does not contain a sub-pulse structure, may appear in complicated way. They are more pronounced if the generated frequencies lie out of the incident pulse spectrum. We also see induced spurious absorption frequencies, which are the linear combinations of various frequencies. They have high similarity with the true absorption frequencies of a substance. This fact allows for us to use both the emission frequencies and the induced spurious absorption frequencies for the detection and identification of substances instead of the true absorption frequencies of a medium. Obviously, it is necessary to take the spectral intensity of generated frequencies into account, which depends on the incident pulse power density, in particular.

## 3. Experimental Setups 

Obviously, from provided consideration the following question arises: how can we verify the effect of the spurious absorption frequency generation in physical experiment? We mentioned above that it could be done by using two types of the physical experiments providing with narrowband and broadband THz signals transmitted through the substance.

In the first experiment, the absorption frequencies of 2,4-DNT sample are investigated. The measurements of a broadband THz pulse transmitted through the sample with 2,4-DNT were made using a Ti:sapphire regenerative amplifier (Spectra-Physics Mai Tai) delivering optical pulses with a duration of 100 fs and a central wavelength of 800 nm at a repetition rate of 1 kHz, with noise less than 0.15% [48,60,61,62]. The transmitted THz signal was detected by free-space electro-optic sampling in a 1-mm-thick <110> ZnTe crystal with the sampling pulse. The DNT sample was 0.5 mm thickness. 

In the second experiment, we investigate the ordinary materials—aspirin, soap, paper, chocolate, and polyethylene bag. The measurements were made using a narrowband CW THz radiation spectrometer Spectra 400 (TeraView Ltd., Cambridge, UK) in the room atmosphere. The spectral range, in which the measurements were provided, is 0.15–1.6 THz, frequency resolution is 100 MHz, signal-to-noise in 100–1000 GHz range is less than 60 db [63]. The frequency step at the measurement is equal to Δ*ν* = 0.001 THz. Obviously, the spectral resolution is the same. The distance between the sample and the receiver does not exceed 20 cm. Below we will call the spectra measured while using the narrowband CW THz radiation as narrowband spectra for brevity.

## 4. Results of the Experiment Using a Broadband THz Pulse 

There are a number of papers in which the substance absorption frequencies were measured in transmission mode using the broadband THz pulse. For example, in [6], the THz spectra of 15 ERCs were measured in transmission mode with a broadband THz pulse and FTIR spectrometer (Bruker IFS 66 V/S) allowing for providing measurements in the 0.1–21 THz frequency range made in a vacuum or a nitrogen atmosphere. Their absorption frequencies of the RDX, HMX, 2,4-DNT, and 2,6-DNT are the following:

RDX: *ν* = 0.82, 1.05, 1.50, 1.96, 2.20, 3.08, 6.73, 10.35, 11.34, 12.33, 13.86, 14.52, 17.74, 18.12, 20.13 THz
HMX: *ν* = 1.78, 2.51, 2.82, 5.31, 6.06, 11.28, 12.00, 12.54, 12.96, 13.74, 14.55, 18.15, 18.60, 19.38 THz;
2,4-DNT: *ν* = 0.45, 0.66, 1.08, 2.52, 4.98, 8.88, 10.56, 11.58, 12.81, 14.34, 15.69, 19.05, 20.04 THz; and,
2,6-DNT: *ν* = 1.10, 1.35, 1.56, 2.50, 5.61, 6.75, 9.78, 11.43, 13.32, 13.89, 15.39, 17.25 THz.

In the spectra of each of these substances, there are the absorption frequencies, which are equal or close to the linear combinations of other ones. Some of these linear combinations contain the doubled absorption frequency. For example, for RDX, one can see the following combinations: *ν* = 13.86 − 12.33 = 1.53 THz (close to 1.50 THz), *ν* = 14.52 − 12.33 = 2.19 THz (close to 2.20 THz), and *ν* = 6.73·2 − 3.08 = 10.38 THz (close to 10.35 THz). For the substance HMX, we see: *ν* = 1.78·2 + 2.51 = 6.07 THz (close to 6.06 THz). Additionally, for 2,4-DNT, there are two exact linear combinations containing the doubled absorption frequency: *ν* = 4.98·2 − 1.08 = 8.88 THz, *ν* = 10.56·2 − 1.08 = 20.04 THz. Note that both linear combinations are formed with the participation of the same absorption frequency *ν* = 1.08 THz. For 2,6-DNT, there are also two linear combinations with the doubled absorption frequency: *ν* = 1.56·2 + 2.5 = 5.62 THz (close to 5.61), *ν* = 11.43·2 − 17.25 = 5.61 THz. The last one is exact. 

**Figure 10:**
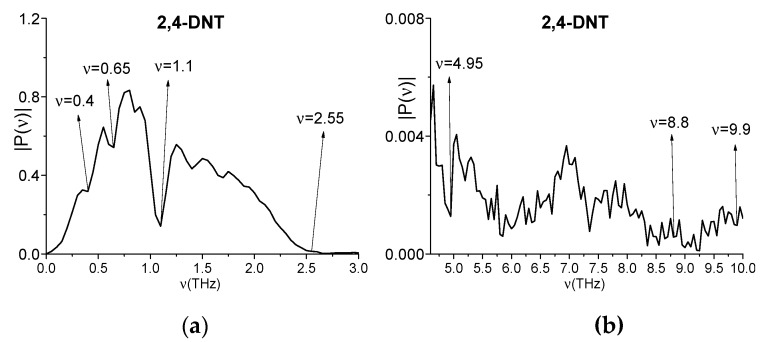

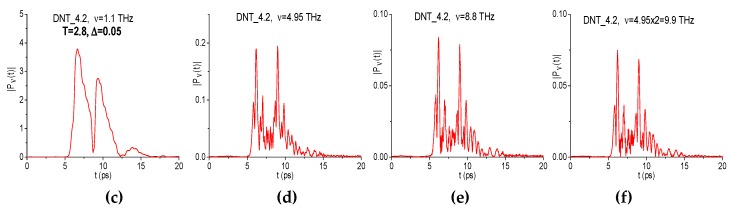
2,4-DNT spectrum in the frequency ranges *ν* = [0, 3.0] THz (**a**) and [4.6, 9.0] THz (**b**). Evolution of the spectral amplitudes modulus at the frequencies *ν* = 1.1 THz (**c**), 4.95 THz (**d**), 8.8 THz (**e**), 9.9 THz (**f**) computed with the window length T = 2.8 ps and window shift *Δ* = 0.05 ps (**c**–**f**)).

In [48] an investigation of the 2,4-DNT detection we used the measurements of the absorption frequencies of 2,4-DNT made in transmission mode at relative humidity 4.2% and in the time interval t = [0, 20] ps with time step $h_{t}$ = 0.05 ps. Because we use this substance as example in current paper then, in Figure 10, we show its spectrum in the frequency ranges *ν* = [0, 3.0] THz (a), [4.6, 9.0] THz (b) made with the spectrum resolution Δ*ν* = 0.05 THz. The spectral minima at the frequencies *ν* = 0.4, 0.65, 1.1, 1.4, 2.55, 4.95, and 8.8 THz are in a good agreement with the absorption peaks mentioned above [6]. It should be stressed that, in the 2,4-DNT spectrum (a), (b) one can also see the linear combination of the frequencies containing the doubled absorption frequency, which is close to the linear combination of frequencies mentioned above: *ν* = 4.95·2 − 1.1 = 8.8 THz. The minimum at the doubled frequency *ν* = 9.9 THz = 4.95·2 THz is also present in (b). Therefore, these measurements are correct.

In (c–f), an evolution of the spectral amplitude modulus at the frequencies *ν* = 1.1, 4.95, 8.8, and 4.95·2 = 9.9 THz are depicted, which is computed with the window length T = 2.8 ps and the window shift Δ = 0.05 ps. 

Appendix B demonstrates the computation of the integral spectral intensity $\left|P_{dyn}(\nu )\right|$ (i.e., the brightness of the spectral line) using the time-dependent spectral brightness. We show that the value of the integral spectral intensity $\left|P_{dyn}(\nu )\right|$ computed at the chosen frequency ν does not depend on the window length T and its shift Δ, and it is equal to the spectral amplitude $\left|P{\left(\nu ,t_{b}\right)}_{L_{t}}\right|$ of the Fourier transform of the function F(t) at this frequency in the time interval $\left[t_{b},t_{e}\right]$ length $L_{t}=t_{e}-t_{b}$. The second remark refers to choosing of the window shift Δ = 0.05 ps. This value follows from a requirement of computation of the spectral dynamics at high frequencies *ν* = 4.95, 8.8, 9.9 THz ≤ *ν*_max_ = 10 THz. According to the Nyquist–Shannon-Kotel’nikov sampling theorem [64,65,66], the maximal frequency, which can be resolved for the time step $h_{t}$, is $\nu_{\max}\leq 1/(2\cdot h_{t})$. At construction of the spectral line dynamics, the window shift Δ plays the role of the time step $h_{t}$. Thus, the window shift Δ must satisfy the condition $\Delta \leq 1/(2\cdot \nu_{\max})$ = 0.05 ps. For convenience, the influence of window shift Δ on the accuracy of constructing the spectral line dynamics is investigated in Appendix C.

We see in Figure 10 that the shapes of the time-dependent spectral amplitudes for the frequencies *ν* = 4.95, 8.8, 9.9 THz are very similar, and the corresponding correlation coefficient are close to unity: $c_{4.95,9.9}$ = 0.95, $c_{4.95,8.8}$ = 0.96, $c_{8.8,9.9}$ = 0.952. The spectral amplitudes of these frequencies are equal to the following values: |P(1.1)| = 0.141, |P(4.95)| = 2.73·10^−3^, |P(8.8)| = 5.72·10^−4^, and |P(9.9)| = 9.69·10^−4^ (Figure 10a,b). Taking into account that the additional absorption frequency *ν* = 8.8 THz is the linear combination of the frequencies *ν* = 4.95, 1.1 THz (*ν* = 8.8 = 4.95·2 − 1.1 THz), it might mean that, during the process of the frequency *ν* = 8.8 THz generation, the part of the pulse energy corresponding to the base frequencies *ν* = 4.95, 1.1 THz can be transferred to the harmonics at the frequency *ν* = 8.8 THz. To clarify this, it is necessary to analyze the beginning of the spectral intensity evolution at these frequencies.

**Figure 11:**
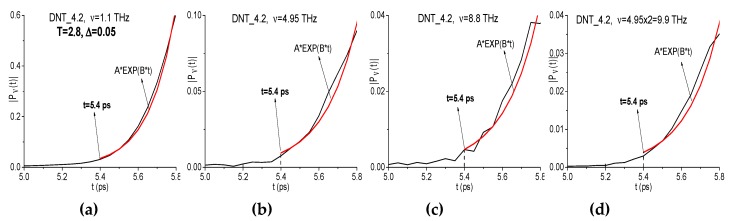
Evolution of the spectral amplitude modulus at the frequencies *ν* = 1.1 THz (**a**), 4.95 THz (**b**), 8.8 THz (**c**), 9.9 THz (**d**) in the time interval t = [5.0, 5.8] ps computed for the 2,4-DNT substance (black line). Red line is an approximating exponential function.

In Figure 11 the magnified view of the evolution of spectral amplitudes is shown in the time interval t = [5.0, 5.8] ps, which corresponds to the beginning of the THz pulse interaction with the 2,4-DNT sample. The spectral line dynamics (a–d) are approximated with exponential function $F_{ap}(t)=a\exp(bt)$. The corresponding coefficient b, which determines the growth rate of a function $F_{ap}(t)$, is equal to 7.26 (a), 5.76 (b), 5.56 (c), and 5.67 (d). Thus, in (c,d), the growth rate of a function $F_{ap}(t)$ as well as of the spectral amplitudes evolution is less than in (a,b). At the frequencies *ν* = 1.1, 4.95 THz (a), (b) the THz pulse interaction with a substance begins at the time moment t = 5.4 ps. This time moment corresponds to the beginning of the exponential spectral amplitude growth. At the same time, at the frequencies *ν* = 8.8 THz and *ν* = 4.95·2 = 9.9 THz (c), (d) the non-zero intensity starts a bit later, at the time moment t = 5.4 ps. Thus, we observe the time delay Δt = 0.2 ps in the beginning of the frequency generation in comparison with the spectral dynamics at the frequency *ν* = 4.95 THz. Such a kind of delay usually corresponds to the doubled frequency *ν* = 4.95·2 = 9.9 THz generation process. 

Therefore, one can see high correlation between the spectral line dynamics at the frequencies *ν* = 4.95 THz and *ν* = 4.95·2 = 9.9 THz. Thus, the time delay at the beginning of the generation at the frequency *ν* = 4.95·2 = 9.9 THz with respect to spectral intensity at the basic frequency *ν* = 4.95 THz, as well as the growth rate of the spectral amplitude evolution at *ν* = 4.95·2 = 9.9 THz, which is less than at *ν* = 4.95 THz, confirm the appearance of a doubled absorption frequency in the 2,4-DNT spectrum due to the frequency conversion process.

**Figure 12:**
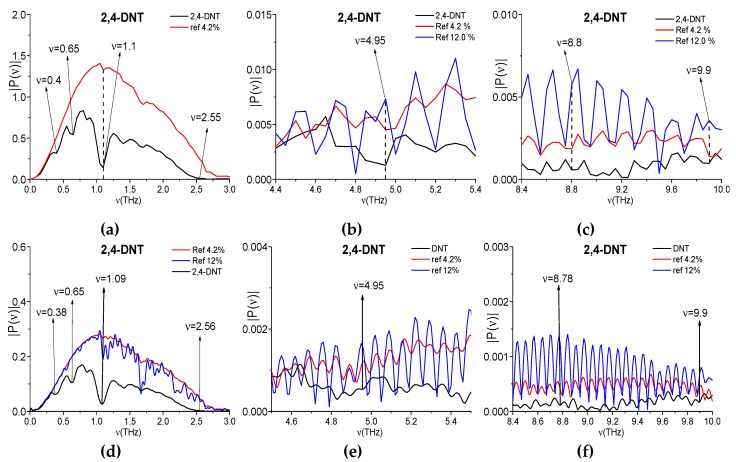
Spectrum of the 2,4-DNT signal measured at 4.2% humidity (black line) and the reference spectra measured at 4.2% humidity (red line) and 12% humidity (blue line) in the frequency ranges *ν* = [0, 3.0] THz (**a**,**d**), [4.6, 5.4] THz (**b**,**e**) and [8.4, 10.0] THz (**c**,**f**). Spectral resolution is equal to Δ*ν* = 0.05 (**a**–**c**), 0.01 ps (**d**–**f**).

Because, in our data of the physical experiments, an influence of humidity is present, we need to discuss this influence on the 2,4 DNT absorption frequencies. For this aim, we compare the absorption frequencies *ν* = 1.1, 4.95, 8.8, and 9.9 THz of the 2,4-DNT signal measured at 4.2% relative humidity with the corresponding values in the spectra of reference signal measured in the ambient air with increasing (from 4.2% up to 12 %) relative humidity. In Figure 12a–c, the spectrum of the 2,4-DNT (4.2% relative humidity) is depicted together with the reference spectra measured at 4.2% (a–c) and 12% (b,c) relative humidity in the frequency ranges containing the frequencies *ν* = 1.1 (a), 4.95 (b), 8.8, and 9.9 THz (c). In (a), at relative humidity 4.2%, the water vapor practically does not influence on the 2,4-DNT spectral minimum at the frequency *ν* = 1.1 THz. In (b,c), one can see the minima at the frequencies *ν* = 4.95, 8.8, and 9.9 THz in the 4.2% humidity reference spectrum (red line). However, these minima are not preserved in the 12% humidity reference spectrum (blue line). 

In (d–f), the same spectra are computed at decreased spectral resolution Δ*ν* = 0.01 ps. One can see that, for both values of humidity 4.2% (red line) and 12% (blue line), the minima at the 2,4-DNT absorption frequencies *ν* = 4.95, 8.8 and 9.9 THz are absent in the reference spectra (e,f). In (d) one can see a minimum at the frequency *ν* = 1.09 THz in the reference spectrum (blue line), but it is weakly pronounced. Thus, an increase of spectral resolution confirms that the minima at the frequencies *ν* = 1.1, 4.95, 8.8, and 9.9 THz in the 2,4-DNT spectrum do not appear because of the THz radiation absorption by water vapor and they are true 2,4-DNT absorption frequencies. 

## 5. Results of the Experiment Using a Narrowband CW THz Radiation

In this Section, we investigate the absorption frequencies that appear at the narrowband THz signal transmission through the neutral substances and compare them with the absorption frequencies in the spectra appearing at the broadband THz signal transmission through the similar substances.

The aim of this consideration is the finding of the absorption frequencies that are absent at measurement with CW THz radiation. Figure 1 shows the scheme of providing of the physical experiment. If a minimum at the doubled absorption frequency in the spectrum of the narrowband THz signal transmitted through the sample is present, then this is a true absorption frequency (Figure 1a). However, the up-conversion process induces this frequency if the spectrum minimum at the doubled frequency is absent at the action of the narrowband THz pulse (Figure 1b). 

Let us notice that the first sample is the standard aspirin pellet, the second one is a piece of soap, and the third one is a standard printer paper. The fourth example is a chocolate bar and powdered chocolate in a polyethylene bag. The empty polyethylene bag is also investigated.

### 5.1. Aspirin Pellet

**Figure 13:**
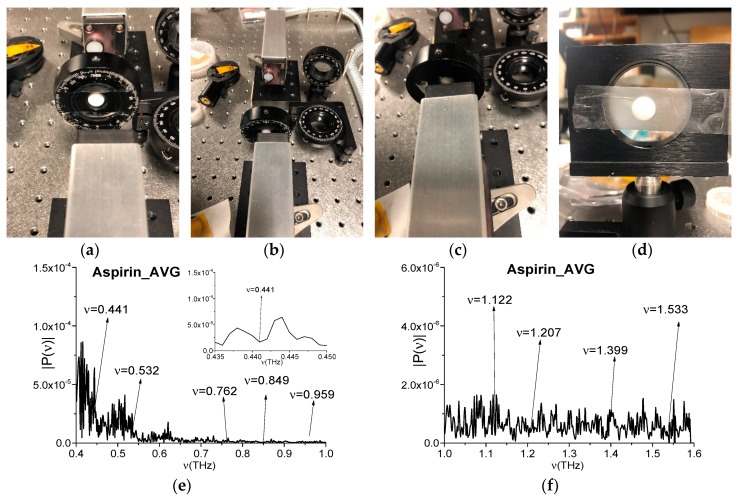

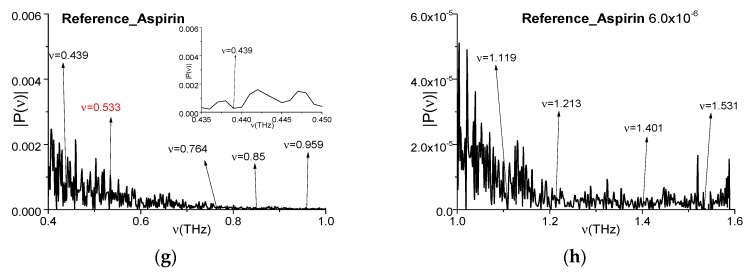
Aspirin pellet in the THz spectrometer (**a**–**d**), Aspirin_AVG spectra (**e**,**f**) and reference spectra (**g**,**h**) in the frequency ranges *ν* = [0.4, 1.0] THz (**e**,**g**) and [1.0, 1.6] THz (**f**,**h**).

In Figure 13a–c, a standard aspirin pellet is shown at different angles to the receiver. In Figure 13d, the pellet is placed in the thin polyethylene bag. The measured narrowband spectra were averaged over a number of measurements (i.e., four) and the resulted spectrum (Aspirin_AVG, for brevity) is depicted in the frequency ranges *ν* = [0.4, 1.0] THz (e) and [1.0, 1.6] THz (f). In accordance with the RIKEN/NICT database [67], the broadband spectrum of Aspirin possesses the following absorption frequencies in the frequency range *ν* < 1.6 THz: *ν* = 0.44, 0.53, 0.76, 0.85, 0.96, 1.12, 1.21, 1.4, and 1.53 THz. In (e), (f), one can see the Aspirin_AVG spectral minima at the frequencies *ν* = 0.441, 0.532, 0.762, 0.849, 0.959, 1.122, 1.207, 1.399, and 1.533 THz, which are equal or close to those from RIKEN database. 

For finding of influence of the water vapor on the measured spectrum, in Figure 13g,h, the reference spectrum is depicted in the same frequency ranges. It contains the spectral minima at the frequencies close to those of Aspirin_AVG Figure 13e,f, but the most part of them lies at the spectral distances exceeding the spectral resolution. Nevertheless, the Aspirin_AVG spectral minima at the frequencies *ν* = 0.532 THz and 0.959 THz in Figure 13e can be caused by environment influence, because, in the reference spectrum Figure 13g, there are spectral minima at the frequencies *ν* = 0.533 THz and 0.959 THz, which are within the spectral resolution.

Let us notice that one of the absorption frequencies of Aspirin, being measured at using the broadband THz pulse (RIKEN), is a linear combination of the basic absorption frequencies, and we name this frequency as *ν*_minus_ = 0.44 THz = (1.4–0.96) THz. For verification, if this absorption frequency is true or spurious one, it is necessary to show the presence (or absence) of such a minimum in the Aspirin_AVG spectrum, measured using the narrowband signal. We see in Figure 13e that there is a minimum at the Aspirin_AVG frequency *ν* = 0.441 THz. However, its appearance might be caused by the environment influence, because there is the minimum at the close frequency *ν* = 0.439 THz in the reference spectrum (Figure 13g). For clearing, the corresponding spectra are shown in Figure 13e,g in magnified view. Therefore, in this case it is difficult to draw an unambiguous conclusion, since there is a reference spectral minimum at the close frequency *ν* = 0.44–0.001 THz.

### 5.2. Piece of Soap 

**Figure 14:**
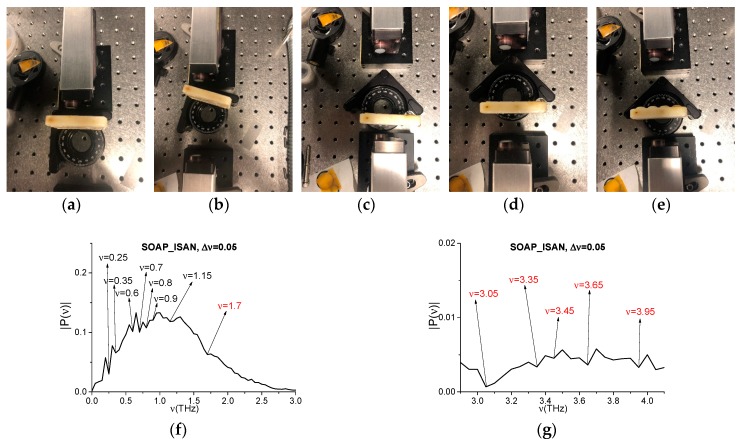

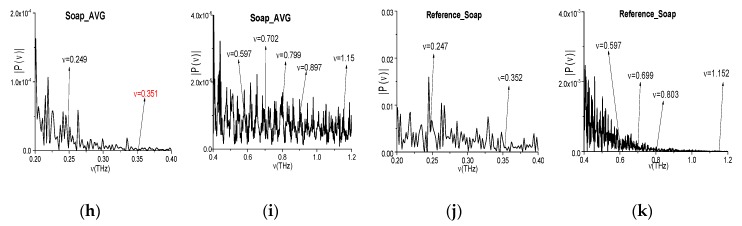
Soap in the THz spectrometer (**a**–**e**), Soap_BB spectrum in the frequency ranges *ν* = [0, 3.0] THz (**f**) and [3.0, 4.0] THz (**g**), Soap_AVG spectrum in the frequency ranges *ν* = [0.2, 0.4] THz (**h**) and [0.4, 1.2] THz (**j**). Reference spectrum (**j**,**k**).

The second example is a piece of soap placed at different distances and angles to the receiver, see Figure 14a–e. In (e), the sample has an inhomogeneous surface. We note that, in [52], we investigated the absorption frequencies of soap (Soap_BB for brevity, BB means “broadband”) in the transmission mode using the broadband THz pulse. Soap_BB spectrum possesses the following absorption frequencies in the frequency range *ν* < 1.6 THz: *ν* = 0.25, 0.35, 0.6, 0.7, 0.8, 0.9, 1.15 THz, as seen in Figure 14f. There are also several spectral minima in the frequency range *ν* > 1.6 THz: *ν* = 1.7, 3.05, 3.35, 3.65, 3.95 THz (f), (g).

**Figure 15:**
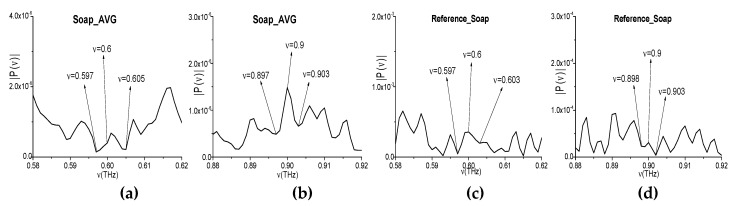
Soap_AVG spectrum (**a**,**b**) and reference spectrum (**c**,**d**) in the frequency ranges *ν* = [0.58, 0.62] THz (**a**,**c**) and *ν* = [0.88, 0.92] THz (**b**,**d**).

The measured spectra using the narrowband THz signals transmitted through the soap samples were averaged over a number of measurements (i.e., five), and the resulting spectrum (Soap_AVG, for brevity) is depicted in the frequency ranges *ν* = [0.4, 1.0] THz (h) and [1.0, 1.6] THz (i). In (h), (i), one can see the Soap_AVG spectral minima at the frequencies *ν* = 0.249, 0.351, 0.597, 0.702, 0.799, 0.897, and 1.15 THz, which are equal or close to those of Soap_BB. We see that, in the Soap_BB spectrum (f), (g) contains the linear combinations of the basic absorption frequencies, including a combination containing a doubled frequency. Thus, these frequencies are *ν*_minus_ = (0.7·2 − 0.8) = (3.95 − 3.35) = (3.65 − 3.05) = 0.6 THz, *ν*_minus_= (3.95 − 3.05) = 0.9 THz. At the same time, in the Soap_AVG narrowband spectrum, the minimum at the frequencies *ν* = 0.6 ± 0.001 THz and *ν* = 0.9 ± 0.001 THz are absent (Figure 15a,b). It is important that, in the reference spectrum, the minima at these frequencies are also absent (Figure 15c,d), and the closest spectral minima are: *ν* = 0.6 ± 0.003 THz (c), 0.9 − 0.002 THz (d). It is far enough, keeping in mind the spectral resolution. Therefore, the appearance of the linear combinations of frequencies *ν*_minus_ = 0.6, 0.9 THz in the spectrum of the broadband pulse Soap_BB is induced by the frequency conversion process. Thus, these frequencies are not the true Soap_BB absorption frequencies.

**Figure 16:**
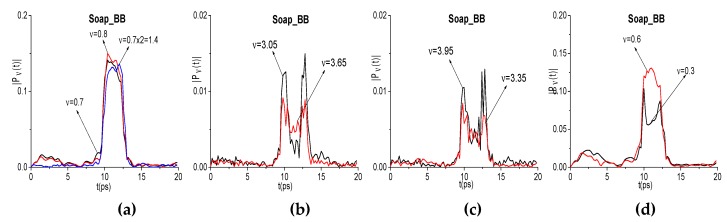
Evolution of Soap_BB spectral amplitude modulus at the frequencies: *ν* = 0.7, 0.8, 1.4 THz (**a**), 3.05, 3.65 THz (**b**), 3.35, 3.95 THz (**c**), and 0.3, 0.6 THz (**d**).

It should be noted that one Soap_BB minimum at the frequency *ν*_minus_ = 0.6 THz corresponds to three different linear combinations of the basic absorption frequencies. Therefore, in Figure 16, we show the evolution of spectral amplitudes at the frequencies: *ν* = 0.7, 0.8, 1.4 = 0.7·2 THz (a), *ν* = 3.05, 3.65 THz (b), and *ν* = 3.35, 3.95 THz (c) that define the frequency *ν* = 0.6 THz generation process. We see that the shapes of the corresponding spectral dynamics at the basic, doubled, and other generated frequencies are similar. The correlation coefficient are very high: $c_{0.7,0.8}$ = 0.997, $c_{0.7,1.4}$ = 0.973, $c_{3.05,3.65}$ = 0.899, and $c_{3.35,3.95}$ = 0.891. 

In our opinion, the appearance of the Soap_BB spectral minima at the frequencies *ν* = 3.35, 3.65, and 3.95 THz (Figure 14g) can be due to the energy levels transitions that are caused by the photons with the frequencies *ν* = 3.05 THz and *ν* = 0.3, 0.6 THz (see the spectral dynamics in (d)), because there is the high correlation between the corresponding spectral line dynamics. The physical mechanism of these energy level transitions is the cascading mechanism that is described in [57,58], which occurs under the action of the broadband THz pulse. We analyzed such a mechanism of the energy level excitation in the framework of one-dimensional (1D) Maxwell’s equations describing the pulse propagation and matrix density formalism for the description of the response of a medium. However, the direct experimental verification of our supposing might be made only using the narrowband CW radiation.

### 5.3. Sheet of Paper 

**Figure 17:**
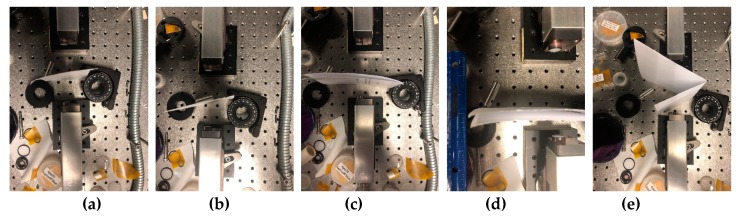
Location of the paper sheets in the THz spectrometer (**a**–**e**). One (**a**,**b**), two (**c**,**d**) and four sheets (**e**) placed at different distances and angles with respect to the receiver.

**Figure 18:**
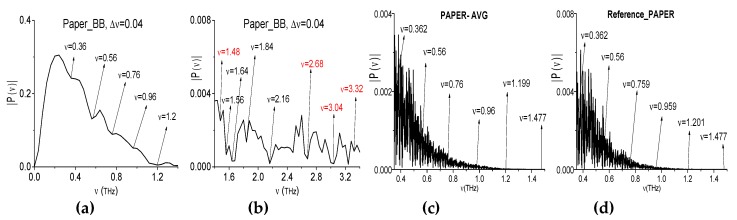
The Paper_BB spectrum in the frequency ranges *ν* = [0, 1.4] THz (**a**), [1.4, 3.4] THz (**b**). The Paper_AVG (**c**) and reference spectrum (**d**) in the frequency range *ν* = [0.35, 1.5] THz.

The third example is a standard sheet of paper. One, two, or four sheets are placed at different distances and angles with respect to the receiver (see Figure 17a–e). We stress that, in [53], the absorption frequencies of the paper sheet (Paper_BB, for brevity) were found out in transmission mode using the broadband THz pulse. They are depicted in the Figure 18a,b for the frequency range *ν* < 1.6 THz *ν* = 0.36, 0.56, 0.76, 0.96, 1.2, and 1.48 THz. For another frequency range *ν* > 1.6 THz they are equal to *ν* = 1.56, 1.64, 1.84, 2.16, 2.68, 3.04, and 3.32 THz. These physical experiment results are necessary for us for comparison of those absorption frequencies with the absorption frequencies measured while using the narrowband THz CW radiation.

The spectra of the narrowband THz signals that were transmitted through the paper samples were averaged over the number of measurements (i.e., six) and the resulted spectrum (Paper_AVG for brevity) is depicted in Figure 18a for the frequency range *ν* = [0.35, 1.5] THz. It possesses the spectral minima at the frequencies *ν* = 0.362, 0.56, 0.76, 0.959, 1.199, and 1.477 THz. One can see that the Paper_AVG spectral minima and the Paper_BB ones are in a good agreement. At the same time, in the reference spectrum (d) one can see the minima at the close or equal frequencies. This circumstance is requires an additional analysis.

**Figure 19:**
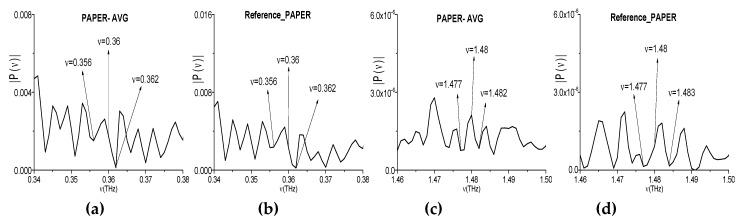
Paper_AVG signal spectrum (**a**,**c**) and reference spectrum (**b**,**d**) in the frequency ranges *ν* = [0.34, 0.38] THz (**a**,**b**) and *ν* = [1.46, 1.50] THz (**c**,**d**).

Note that, in the spectrum of the broadband Paper_BB signal (Figure 18a,b), one can observe the frequencies, which are linear combinations of the basic absorption frequencies. One of them contains a doubled absorption frequency. For example, we point out the frequencies *ν*_minus_ = (0.96·2 − 1.56) = (3.04 − 2.68) = 0.36 THz; *ν*_minus_ = (2.68 − 1.2) = (3.32 − 1.84) = 1.48 THz. At the same time, in the Paper_AVG spectrum the minima at the frequencies *ν* = 0.36 ± 0.001 THz and *ν* = 1.48 ± 0.001 THz are absent (see Figure 19). The minima, which are closest to these values, are located at the frequencies *ν* = 0.36 + 0.002 THz (a) and *ν* = 1.48 ± 0.003 THz (c). It is very important that the minimum at the frequencies *ν* = 0.36 ± 0.001 THz and *ν* = 1.48 ± 0.001 THz are also absent in the reference spectrum (Figure 19b,d). 

Therefore, the frequency conversion process causes the appearance of the frequencies *ν*_minus_ = 0.36 THz and 1.48 THz, which are linear combinations of absorption frequencies, in the spectrum of the broadband Paper_BB signal.

### 5.4. Polyethylene Bag

**Figure 20:**
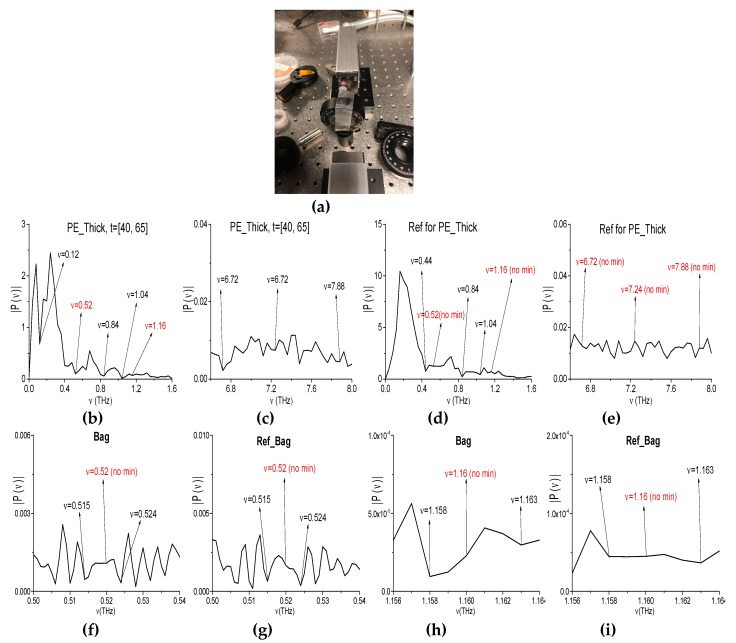
Empty polyethylene bag (**a**) in the THz setup. The spectrum of the broadband PE_Thick pulse (**b**,**c**) and the reference spectrum (**d**,**e**) in the frequency ranges *ν* = [0, 1.6] THz (**b**,**d**) [6.6, 8.0] THz (**c**,**e**). The spectrum of the narrowband Bag signal (**f**,**h**) and the reference spectrum (**g**,**i**) in the frequency ranges *ν* = [0.5, 0.54] THz (**f**,**g**), [1.156, 1.164] THz (**h**,**i**).

Below, we investigate the spectral properties of the empty polyethylene bag (Figure 20a) using a narrowband THz pulse (Bag signal, for brevity). The spectral properties of polyethylene are well-known. Accordingly, with accordance to RIKEN THz database [67], the polyethylene (PE) does not demonstrate any pronounced absorption frequencies in the frequency range *ν* < 3.0 THz, but, in the frequency range *ν* > 3.0 THz, it possesses two frequencies: *ν* = 6.72, 7.88 THz. In [59], we investigated the broadband THz pulse at non-zero humidity. In Figure 20, the corresponding spectrum of the first sub-pulse of the THz signal reflected from a pure polyethylene (PE_Thick, for brevity) is shown in the frequency ranges *ν* = [0, 2.0] THz (b) and [6.4, 8.0] THz (c). In (d), (e), the reference spectrum is depicted in the same frequency ranges. These measurements were made with the spectral resolution Δ*ν* = 0.04 THz. 

When comparing PE_Thick and reference spectra in the range *ν* = [0, 2.0] THz (b), (d), one can see that the part of spectral minima is caused by the environment or noise influence. Only two spectral minima at the frequencies *ν* = 0.52, 1.16 THz (b) are free from this influence, because the corresponding spectral minima in (d) are absent. In the frequency range *ν* = [6.4, 8.0] THz (c) one can see two minima *ν* = 6.72, 7.88 THz, which are equal to the PE absorption frequencies (RIKEN). Let us note that the minima at these frequencies in the reference spectrum (e) are also absent. Once more minimum in the PE_Thick spectrum, which is not caused by the environment or noise, is located at the frequency *ν* = 7.24 THz (c), (e). Additionally, the spectral minima in (b) at the frequencies *ν* = 0.52, 1.16 THz are the caused by the frequency conversion process for appearance of the broadband PE_Thick absorption frequencies: *ν*_minus_ = (7.24 − 6.72) =0.52 THz; and, *ν*_minus_ = (7.88 − 6.72) = 1.16 THz. 

For clarity, in (f), (h), the spectrum of the narrowband Bag signal is depicted in the frequency ranges *ν* = [0.5, 0.54] THz (f), [1.156, 1.164] THz (h). The spectral minima at the frequencies *ν* = 0.52 THz and 1.16 THz are absent in (f), (h), which refers to the narrowband Bag signal spectrum. Taking the spectral resolution Δ*ν* = 0.001 THz and the absence of the corresponding minima in the reference spectrum (g), (i) into account, we conclude that an appearance of these frequencies *ν*_minus_ = 0.52 THz and 1.16 THz in the spectrum of the broadband PE_Thick pulse (Figure 20b,c) is caused by the frequency conversion process. Therefore, these frequencies are spurious ones in the PE_Thick pulse spectrum.

### 5.5. Chocolate Bar

**Figure 21:**
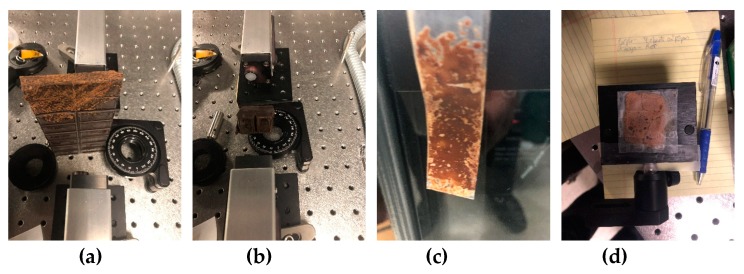
Samples with chocolate bar (**a**,**b**) and powdered chocolate in the polyethylene bag (**c**,**d**) in the THz setup.

**Figure 22:**
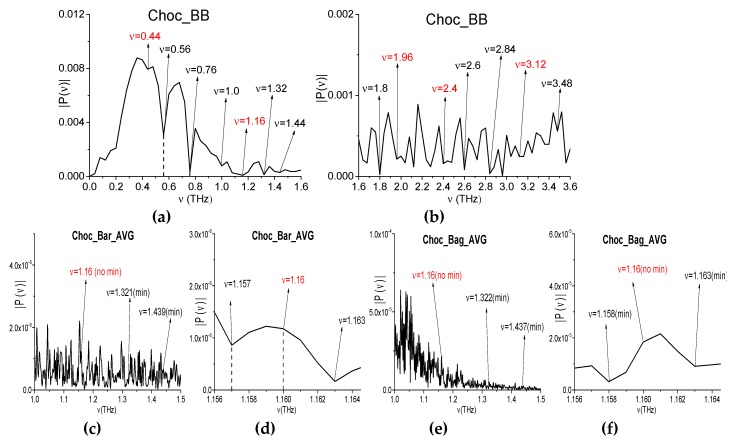

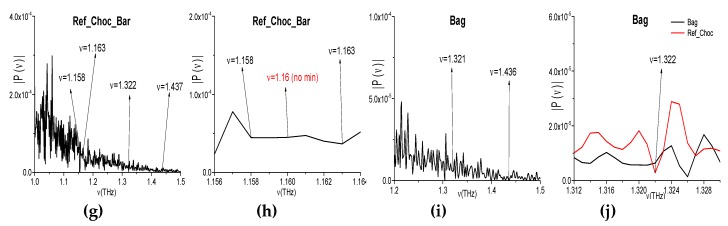
Spectrum of the Choc_BB signals measured using the broadband THz signal (**a**,**b**) in the frequency ranges *ν* = [0, 1.4] THz (**a**), [1.6, 3.2] THz (**b**). Spectra of the Choc_Bar_AVG signals (**c**,**d**) and Choc_Bag_AVG signals (**e**,**f**) measured using the narrowband THz signal in the frequency ranges **ν** = [1.0, 1.5] THz (*c*,**e**) and [1.156, 1.164] THz (**d**,**f**). Reference spectrum (*g*,**h**) in the same frequency ranges. Bag spectrum in the frequency range *ν* = [1.2, 1.5] THz (**i**), *ν* = [1.312, 1.328 THz (**j**).

In the last example, we consider the samples with chocolate bar and powdered chocolate in the polyethylene bag (see Figure 21a–d). While using the broadband THz pulse in [46], we investigated the absorption frequencies of chocolate (Choc_BB, for brevity) measured in transmission mode. The Choc_BB pulse spectrum possesses the following pronounced spectral minima in the frequency range *ν* < 1.6 THz: *ν* = 0.44, 0.56, 0.76, 1.0, 1.16, 1.32, and 1.44 THz (Figure 22a) and several spectral minima in the frequency range *ν* > 1.6 THz: *ν* = 1.8, 1.96, 2.4, 2.6, 2.84, 2.96, 3.12, and 3.48 THz (b). The spectral resolution in (a), (b) is equal to Δ*ν* = 0.04 THz. In those experiments, the obtained spectral minima at the frequencies *ν* = 0.56, 0.76, 1.0 THz appear due to water vapor absorption. A part of the spectral minima *ν* = 1.44, 1.8, 2.6, 2.84 THz (a), (b) are close to the absorption frequencies of pure Sucrose: *ν* = 1.43, 1.82, 2.55, 2.8 THz (RIKEN [67]). Therefore, these spectral minima are due to the presence of sugar in the sample.

The spectra of the narrowband THz signals were averaged over the measurements (two for both the chocolate bar and the powdered chocolate). We will call these signals Choc_Bar_AVG and Choc_Bag_AVG, for brevity. In Figure 22 (the averaged spectra of the Choc_Bar_AVG signal (c), (d) and Choc_Bag_AVG signal (e), (f) are shown in the frequency ranges *ν* = [1.0, 1.5] THz (c), (e) and [1.156, 1.164] THz (d), (f). The reference spectrum is depicted in (g), (h) in the same frequency ranges. The spectral resolution in (c)–(h) is equal to Δ*ν* = 0.001 THz.

The Choc_Bar_AVG signal and Choc_Bag_AVG signal spectra demonstrate the spectral minima at the frequencies *ν* = 1.321, 1.439 THz (c), and *ν* = 1.322, 1.437 THz (e), which are in a good agreement with the spectral minima of the broadband Choc_BB signal (a). However, in the reference spectrum (g), one can see the spectral minima at the close or equal frequencies *ν* = 1.322, 1.437 THz. Taking the spectral resolution Δν = 0.001 THz into account means that, in both spectra (c), (e) the spectral minima at the frequencies *ν* = 1.321 THz (c) (1.322 THz (e)) is due the atmospheric air influence. The same situation occurs at the spectral minimum *ν* = 1.437 THz in the Choc_Bag_AVG signal spectrum. 

In Figure 22i, the spectrum of the Bag narrowband THz signal is shown in the frequency range *ν* = [1.2, 1.5] THz. One can see two spectral minima at the frequencies *ν* = 1.321, 1.436 THz, which are also close (within the spectral resolution) to the Ref_Choc_Bar spectral minima at the frequencies ν= 1.322, 1.437 THz (g). Taking the absence of polyethylene bag absorption frequencies in the frequency range *ν* < 3.0 THz (RIKEN database [67]) into account, the appearance of these two minima in the signal Choc_Bag_AVG spectrum can also be caused by the influence of the atmospheric air. That is, the spectral minimum in the signal Choc_Bar_AVG spectrum *ν* = 1.439 THz (c) can be regarded as an absorption frequency of Chocolate. 

In the broadband Choc_BB spectrum Figure 22 (a), (b), one can see the absorption frequencies, which is caused by the frequency conversion process: *ν*_minus_ = (2.96 − 1.8) = (3.12 − 1.96) = 1.16 THz. At the same time, in the Choc_Bar_AVG and Choc_Bag_AVG narrowband spectra this minimum is absent (Figure 22d,f). Moreover, in the reference spectrum (Figure 22h), the minimum at this frequency is also absent. Therefore, this absorption frequency *ν*_minus_ = 1.16 THz is a spurious one observing in the spectrum of the broadband Choc_BB signal that was measured in the transmission mode.

**Figure 23:**
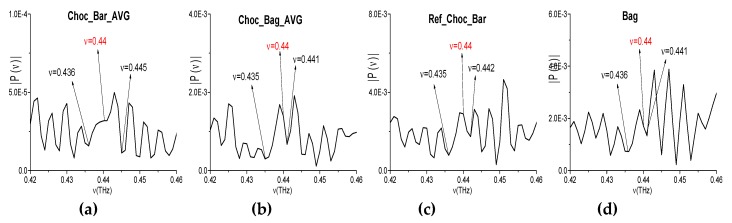
Spectra of the narrowband Choc_Bar_AVG (**a**), Choc_Bag_AVG (**b**) signals and the reference spectrum (**c**), and the Bag one (**d**) in the frequency range *ν* = [0.42, 0.46] THz.

Other possible spurious absorption frequency, including a doubled absorption frequency, is the following: *ν*_minus_ = (1.96·2 − 3.48) = (2.4 − 1.96) = 0.44 THz (Figure 22a,b). The corresponding spectral minimum at this frequency is absent in the spectrum of the narrowband Choc_Bar_AVG signal and in the reference spectrum (Figure 23a,c). Note that water vapor absorption is absent at the frequency *ν* = 0.44 THz [6,67]. Thus, the frequency *ν*_minus_ = 0.44 THz is not a true absorption frequency that belongs to the broadband Choc_BB spectrum. At the same time, in the Choc_Bag_AVG and Bag spectra (b), (d) there is a spectral minimum at the frequency *ν* = 0.441 THz = 0.44 + 0.001 THz. Taking the spectral resolution Δ*ν* = 0.001 THz into account, this minimum in the Choc_Bag_AVG signal spectrum can be caused by the noise influence in this experiment. Therefore, we believe that a conclusion regarding the spurious absorption frequency is right. 

## 6. Conclusions

One of the physical mechanisms of the spurious absorption frequencies appearance caused by the frequency conversion process (the doubled frequency, sum frequency generation, and difference frequency generation) because of the nonlinear response of a medium at the broadband THz pulse interaction with a medium is predicted while using the computer simulation results. The results of a few physical experiments provided with the narrowband THz CW radiation and broadband THz pulses transmitted through the ordinary and dangerous materials confirm this phenomenon. They are more pronounced in the frequency range not belonging to the incident pulse spectrum.

Based on computer simulation results, we show that, if the pulse consists of the intersecting sub-pulses with substantially overlapping spectra, then it is possible to observe the shift of the spurious absorption frequency. At the same time, the dynamics of the spectral lines at the basic frequency and the shifted spurious absorption frequency are shown to be very similar. The correlation between them ranges from 0.88 to 0.98. Let us notice that such a structure of the THz pulse corresponds to its transmission through a layer of a medium (tablet, for example). If the pulse possesses the sub-pulse structure, which corresponds to consideration of the main THz pulse only, then the frequency shift at the conversion process is not observed. The same processes also occur for the emission frequency conversion.

The spurious absorption (emission) frequency appearance might be used as an additional effective tool for the detection and identification of the substance based on the integral correlation criteria due to the high degree of similarity between the spectral line dynamics at the basic and converted frequencies. This method can be especially effective if noise or environment influence mask the basic absorption frequency. 

It is desirable to conduct a physical experiment under laboratory conditions, where there is no influence of water vapor and other factors, in order to illustrate the presence of spurious frequencies in measured THz signals and as possible future direction of the research. In this article, we used the results of the physical experiments conducted under real conditions to emphasize the presence of the discussed effect in this case, which are most important for a practice, and the possibility of its use for the substance detection and identification. We also note that the effect of the carrier frequency shift, which occurs if the THz signal consists of several pulses with overlapping, must be taken into account in both the diagnostics of the substances and in the construction of the expert systems based on artificial intelligence. 

The approach that was developed by us (analysis of the spectral line dynamics) can be used under real conditions together with other THz sensors platforms (for example, [68,69,70,71]) used for the identification and detection of the substance. It might essentially increase a probability of the substance detection. Let us remind that, in contrast to the standard THz TDS method, the analysis of the spectral line dynamics allows for us to identify the substance using a noisy THz signal or in the case of inhomogeneous sample surface, or at an analysis of the mixture of substances with similar spectral properties.

## Figures and Tables

**Table 1:** Parameters of the signal $E_{inc}(t)$ with $\nu_{1}=0.8$, $\nu_{2}=1.2$, $\nu_{3}=1.4$, $\nu_{4}=1.8$.

Example	$A_{k1}$	$A_{k2}$	$A_{k3}$	$A_{k4}$	$t_{k1}$	$t_{k2}$	$t_{k3}$	$t_{k4}$	$\tau_{k1}$	$\tau_{k2}$	$\tau_{k3}$	$\tau_{k4}$
1	4	4	1	1	10	25	42	55	8	2	16	8
2	1	0.8	0.4	0.2	10	14	20	25	1	2	4	1

**Table 2:** Correlation coefficients $c_{\nu_{base},\nu_{dif}}$ between the basis frequency *ν* = $v_{base}$ and the difference frequency *ν* = $\nu_{dif}$ or the sum frequency *ν* = $\nu_{sum}$. Empty squares mean that the corresponding values of the correlation coefficient were not computed.

		Example 1				Example 2			
	$v_{base}$	0.8	1.2	1.4	1.8	0.8	1.2	1.4	1.8
$\nu_{dif},\nu_{sum}$									
0.4		0.565	0.884	0.796	0.345	0.955	0.642	0.448	0.54
0.6		0.839	0.688	0.718	0.418	0.886	0.732	0.576	0.63
2.2		-	-	-	-	0.508	-	0.718	-
3.2		-	-	0.66	0.5	-	-	-	-

**Table 3:** Correlation coefficients $c_{\nu_{base},2\nu_{base}}$ between the basis frequency and the doubled frequency.

	Example 1			Example 2		
$\nu_{base}$	1.345	1.46	1.675	1.06	1.31	1.53
$\approx 2\nu_{base}$	2.74	2.86	3.34	2.1	2.67	3.05
$c_{\nu_{base},2\nu_{base}}$	0.9	0.9	0.79	0.94	0.97	0.93

**Table 4:** Correlation coefficients $c_{\nu_{base},\nu_{add}}$ between the basic frequency $\nu_{base}$ and the doubled frequency $\nu_{add}$.

$\nu_{base}$	0.5	0.8	0.8	0.8	0.5	0.8
$\nu_{add}$	0.2	0.2	1.4	1.8	0.95	1.6
$c_{\nu_{base},\nu_{add}}$	0.966	0.925	0.923	0.916	0.904	0.922

**Figure A1:**
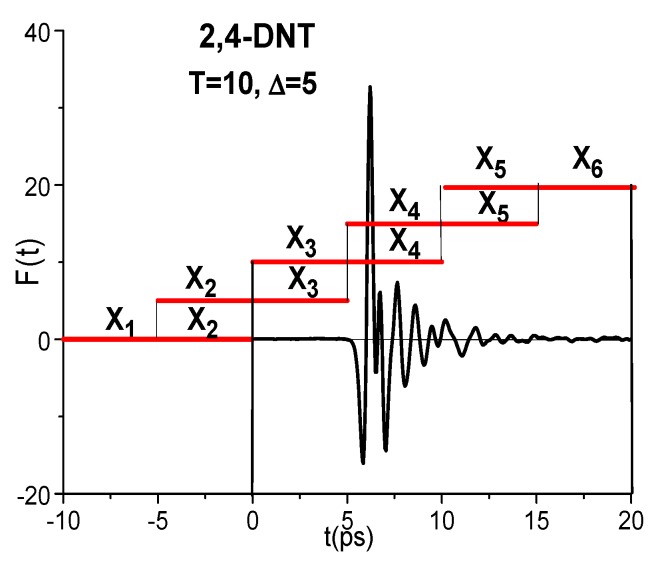
Schematic representation of window length T = 10 ps moving along the 2,4-DNT signal with window shift Δ = 5 ps in the time interval t = [0, 20] ps.

**Figure A2:**
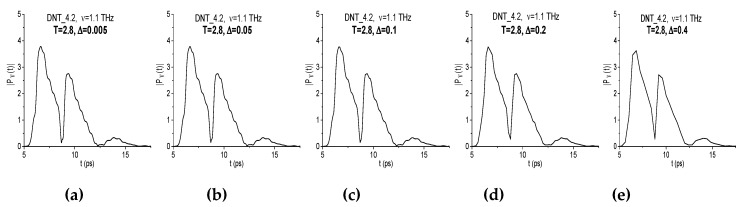
The spectral line dynamics at the frequency *ν* = 1.1 THz computed at the window length T = 2.8 ps and the window shift Δ = 0.005 (**a**), 0.05 (**b**), 0.1 (**c**), 0.2 (**d**), 0.4 (**e**) ps.

**Figure A3:**
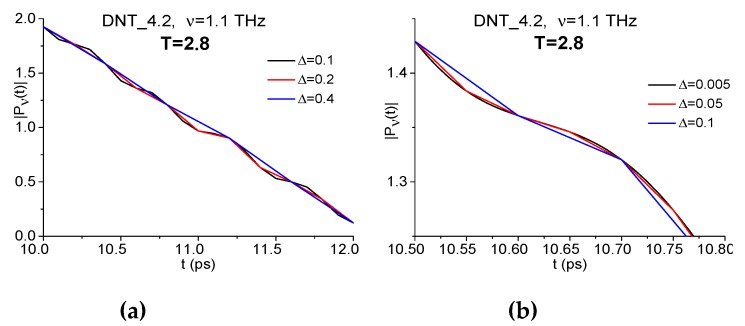

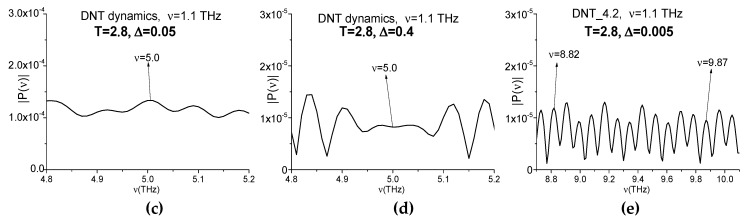
Spectral line dynamics at the frequency *ν* = 1.1 THz computed at the window length T = 2.8 ps and at increasing window shift Δ = 0.1, 0.2, 0.4 ps (**a**), 0.1, 0.05, 0.005 ps (**b**). Spectra of the spectral line dynamics computed at the window shift Δ = 0.05 ps (**c**), 0.4 ps (**d**) and 0.005 ps (**e**).

**Figure A4:**
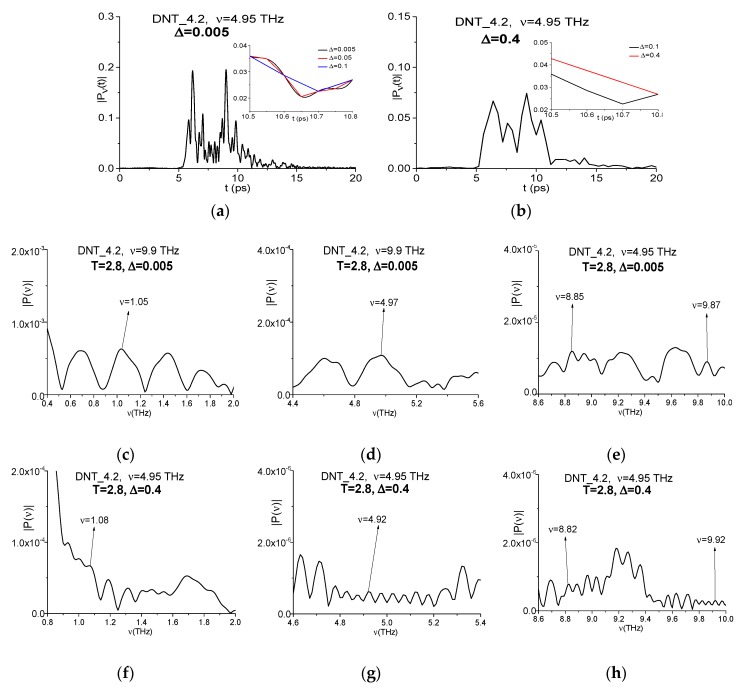
2,4-DNT spectral line dynamics computed at the frequency *ν* = 4.95 THz at the window shift Δ = 0.005 ps (**a**) and 0.4 ps (**b**) (with magnified view). Spectra of the spectral line dynamics computed at the window shift Δ = 0.005 ps (**c**–**e**), 0.4 ps (**f**–**h**) in the frequency ranges *ν* = [0.4, 2.0] THz (**c**), [0.8, 2.0] THz (**f**), [4.4, 5.6] THz (**d**), [4.6, 5.4] THz (**g**), and [8.6, 10.0] THz (**e**,**l**).

## Appendix A. Fourier–Gabor Window Transform

Below we briefly remind the main notations and formulas at the construction of the evolution of the spectral amplitude (dynamics of spectral line) of the signal F(t) at the chosen frequency *ν* [44,45,46].

Let F(t) be the transmitted or reflected THz signal, which is measured in the time interval $\left[t_{b},t_{e}\right]$ length $L_{t}=t_{e}-t_{b}$, where $t_{b}$ and $t_{e}$ denote its beginning and the end. In order to get information about the evolution of the spectral amplitude at the frequency *ν* in the time interval $\left[t_{b},t_{e}\right]$, we use the time window length $T\leq L_{t}$, which moves along the signal. At each step, the time window is shifted on the chosen time interval Δ (window shift). To avoid the spectrum “spreading”, in each window length $L_{t}$ the signal F(t) is multiplied by window function g(t), which quickly tends to zero at the left and right ends of the window. Thus, the Fourier-Gabor transform is carried out in each time window:

(A1) $$P(\nu ,t_{j})=\frac{1}{T}\int_{t_{j}}^{t_{j}+T}F(t)\cdot g\left(t\right)e^{-i2\pi \nu (t-t_{j})}dt,\;g(t)=e^{-{\left(\frac{t-t_{c}}{0.5T}\right)}^{k}},$$

where $t_{j}$ is a time of window beginning, j is a serial number of window, *ν* is a frequency of interest. The units of $t_{j}$, T, Δ and *ν* are measured in ps and THz, respectively. In order to align the beginning of the physical THz pulse and its representation in the evolution of the spectral amplitude under construction, the spectral amplitude value $\left|P_{\nu }(t_{j})\right|$ in the j-th time window is associated with the end of this window:

(A2) $$|P_{\nu }(t_{j})|=|P(\nu ,t_{j}+T)|.$$

To start the computations, the first window is placed in time interval $\left[t_{b}-T,t_{b}\right]$ and the function F(t) is continued by zeros to the left to point $t_{b}-T$. The last window is placed in the time interval $\left[t_{e}-T,t_{e}\right]$. The integrals (A1) are computed using trapezoid method with time step $h_{t}$ = 0.001 ps.

## Appendix B. Computation of the Integral Spectral Intensity Using the Time-Dependent Spectral Brightness

As it is well-known, the spectral intensity at the chosen frequency ν is equal to quadratic power from the Fourier harmonic of the function F(t) at this frequency in this time interval. However, we use the Fourier-Gabor transform at a computation of the spectral line dynamics with a sliding window. Obviously, parts of the signal can be involved many times at its computation. Therefore, there is a question about relation between the spectral intensity of the Fourier transform and the spectral intensity determined from the Fourier-Gabor transform and how does this spectral intensity get. Below we analyze this problem. We show that the spectral intensity does not depend on the window length T and its shift Δ, and it is equal to the spectral intensity of the Fourier harmonic computed on the time interval $\left[t_{b},t_{e}\right]$ under analysis. The result, obtained in this Appendix, is used in Section 4 to estimate the contribution of the pulse energy corresponding to the doubled frequency harmonic in the process of the frequency conversion.

Obviously, if we choose in (A1) the following values of window parameters: $t_{j}=t_{b}$, $T=L_{t}$ and the window function $g(t)=\{\begin{array}{cc} 1.0, & t\in [t_{b},t_{e}]\\ 0, & t\notin [t_{b},t_{e}] \end{array}$, we get the Fourier transform of the signal F(t) in the time interval $\left[t_{b},t_{e}\right]$ at the frequency *ν*:

(A3) $$P{\left(\nu ,t_{b}\right)}_{L_{t}}=\frac{1}{L_{t}}\int_{t_{b}}^{t_{e}}F(t)\cdot e^{-i2\pi \nu (t-t_{b})}dt,$$

and, therefore, the spectral amplitude at the frequency ν is equal to $\left|P{\left(\nu ,t_{b}\right)}_{L_{t}}\right|$ in the time interval $\left[t_{b},t_{e}\right]$. Further, for simplicity, we assume that $t_{b}=0$ and the window function g(t) has the form

$$g(t)=\{\begin{array}{cc} 1.0, & t\in [t_{j},t_{j+T}]\\ 0, & t\notin [t_{j},t_{j+T}] \end{array}.$$

Then from expression (A3) we have:

(A4) $$\int_{0}^{L_{t}}F(t)\cdot e^{-i2\pi \nu t}dt=P{\left(\nu ,0\right)}_{L_{t}}\cdot L_{t}.$$

Let define $N_{W}=T/\Delta$ - the number of shifts Δ in the time window length T, and $N_{D}$ is a number of the window shifting along the signal F(t) from the time point $t_{b}=0$ till the time moment $t_{e}=L_{t}$ with window shift Δ to cover the time interval [0, $L_{t}$]. Thus, one can write $L_{t}=\Delta \cdot (N_{D}-1)$. Then $t_{j}=-T+(j-1)\cdot \Delta$, $j=1,\ldots ,N_{D}$. Therefore, the integral value $P(\nu ,t_{j})$ defined in (A1) can be represented as a sum of integrals over the interval $\left[t_{j},t_{j}+T\right]$:

(A5) $$P(\nu ,t_{j})=\frac{1}{T}\sum_{k=1}^{N_{W}}(\int_{t_{j}+(k-1)\cdot \Delta }^{t_{j}+k\cdot \Delta }F(t)\cdot e^{-i2\pi \nu (t-t_{j})}dt),j=1,\ldots ,N_{D}.$$

From expression (A5) we obtain the following set of linear equations

(A6) $$\sum_{k=1}^{N_{W}}(\int_{t_{j}+(k-1)\cdot \Delta }^{t_{j}+k\cdot \Delta }F(t)\cdot e^{-i2\pi \nu t}dt)=P(\nu ,t_{j})Te^{-i2\pi \nu t_{j}},j=1,\ldots ,N_{D}.$$

with respect to variables $X_{n}$:

(A7) $$X_{n}=\int_{t_{n}}^{t_{n}+\Delta }F(t)\cdot e^{-i2\pi \nu t}dt,n=1,\ldots ,N_{D}+N_{W}-1.$$

The schematic representation of the window moving along the signal F(t) is shown in Figure A1, where the window length and the window shift is equal to *T* = 10 ps and Δ = 5 ps, respectively. A time interval is equal to $L_{t}$ = $\left[t_{b},t_{e}\right]$ = [0,20] = 20 ps. A number of the window shifts Δ covering the time window is equal to $N_{W}=T/\Delta$ = 2. The parameter $N_{D}$ is equal to 5.

The set of equations (A6) has the following form:

(A8) $$\begin{array}{ll} X_{1}+X_{2}+\ldots +X_{N_{W}}=P(\nu ,t_{1})Te^{-i2\pi \nu t_{1}}, & j=1,\\ X_{2}+X_{3}+\ldots +X_{N_{W}+1}=P(\nu ,t_{2})Te^{-i2\pi \nu t_{2}}, & j=2,\\ \ldots \ldots \ldots \ldots \ldots \ldots \ldots \ldots \ldots \ldots \ldots \ldots \ldots \ldots \\ X_{N_{D}}+X_{N_{D}+1}+\ldots +X_{N_{D}+N_{W}-1}=P(\nu ,t_{N_{D}})Te^{-i2\pi \nu t_{N_{D}}}, & j=N_{D}. \end{array}$$

The system (A8) has $N_{D}$ equations with $N_{D}+N_{W}-1$ variables. As we mentioned above, the THz signal F(t) is continued by zeros to the left till the time moment $t_{1}=-T$ in order to start the computation of the spectral amplitude evolution. Thus, we can assume that the following equations

$$X_{1}=0,X_{2}=0,\ldots ,X_{N_{W}-1}=0$$

are valid. Let us note if the value F(0)≠0, then we put the value $X_{N_{W}}=F(0)/2$. Thus, a number of the variables in the set of equations (A8) becomes equal to the number of the equations, and they take the form:

(A9) $$\begin{array}{ll} X_{N_{W}}=P(\nu ,t_{1})Te^{-i2\pi \nu t_{1}}, & j=1,\\ X_{N_{W}}+X_{N_{W}+1}=P(\nu ,t_{2})Te^{-i2\pi \nu t_{2}}, & j=2,\\ \ldots \ldots \ldots \ldots \ldots \ldots \ldots \ldots \ldots \ldots \ldots \ldots \ldots \ldots \\ X_{ND}+X_{ND+1}+\ldots +X_{ND+N_{W}-1}=P(\nu ,t_{ND})Te^{-i2\pi \nu t_{ND}}, & j=ND. \end{array}$$

The matrix of the system (A9) is lower-triangular one with units on the main diagonal. Accordingly, the set of equations has a unique solution:

(A10) $$\begin{array}{ll} X_{N_{W}}=P(\nu ,t_{1})Te^{-i2\pi \nu t_{1}}, & j=1,\\ X_{N_{W}+1}=P(\nu ,t_{2})Te^{-i2\pi \nu t_{2}}-X_{N_{W}}, & j=2,\\ \ldots \ldots \ldots \ldots \ldots \ldots \ldots \ldots \ldots \ldots \ldots \ldots \ldots \ldots \\ X_{N_{D}+N_{W}-1}=P(\nu ,t_{N_{D}})Te^{-i2\pi \nu t_{N_{D}}}-\sum_{k=1}^{N_{W}-1}X_{N_{D}+k-1}, & j=N_{D}. \end{array}$$

Our next step consists in finding the sum of variables $X_{1}$, $X_{2}$, …,$X_{N_{W}}$, $X_{N_{W}+1}$, $X_{N_{W}+2}$, …, $X_{N_{D}+N_{W}-1}$, which gives contribution to the integral spectral amplitude $\left|P_{dyn}(\nu )\right|$ at the chosen frequency ν in the time interval $\left[0,L_{t}\right]$. It can be found directly using expressions (A10). Indeed, taking into account that $X_{1}=0$, $X_{2}=0$, …, $X_{N_{W}-1}=0$ and $X_{N_{W}}$ does not contribute to the integral spectral amplitude $\left|P_{dyn}(\nu )\right|$ because it is computed in the time interval $\left[-\Delta ,0]\notin [0,L_{t}\right]$ (in Figure A1 this is denoted as $X_{2}$), we get:

(A11) $$|P_{dyn}(\nu )|=\frac{1}{L_{t}}|\sum_{n=N_{W}+1}^{N_{D}+N_{W}-1}X_{n}|.$$

Further, the expression (A11) can be transformed to the simpler one using the expressions (A7), (A3):

(A12) $$\begin{array}{l} |P_{dyn}(\nu )|L_{t}=|\sum_{n=N_{W}+1}^{N_{D}+N_{W}-1}X_{n}|=|\sum_{n=N_{W}+1}^{N_{D}+N_{W}-1}\int_{t_{n}}^{t_{n}+\Delta }F(t)\cdot e^{-i2\pi \nu t}dt|=\\ =|\int_{0}^{t_{e}=L_{t}}F(t)\cdot e^{-i2\pi \nu t}dt|=|P{\left(\nu ,0\right)}_{L_{t}}|\cdot L_{t}. \end{array}$$

Thus, the integral spectral amplitude can be found from (A12):

(A13) $$|P_{dyn}(\nu )|=\frac{1}{L_{t}}|(\sum_{n=N_{W}+1}^{N_{D}+N_{W}-1}X_{n})|=\frac{1}{L_{t}}|(P{\left(\nu ,0\right)}_{L_{t}}\cdot L_{t})|=|(P{\left(\nu ,0\right)}_{L_{t}}|=\frac{1}{L_{t}}|\int_{0}^{L_{t}}F(t)\cdot e^{-i2\pi \nu t}dt|.$$

It is easy to see that the same result occurs if $t_{b}\neq 0$:

(A14) $$\left|P_{dyn}(\nu )|=|(P{\left(\nu ,t_{b}\right)}_{L_{t}}|=\frac{1}{L_{t}}|\int_{t_{b}}^{t_{e}}F(t)\cdot e^{-i2\pi \nu (t-t_{b})}dt\right|$$

We should emphasize an important conclusion following from expressions (A13), (A14). Namely, the integral spectral amplitude $\left|P_{dyn}(\nu )\right|$, which is found from the set of equations (A9), does not depend on the window length T and its shift Δ and it is equal to the spectral amplitude $\left|P{\left(\nu ,t_{b}\right)}_{L_{t}}\right|$ of the Fourier transform of the function F(t) at the frequency ν in the time interval $\left[t_{b},t_{e}]=[t_{b},t_{b}+L_{t}\right]$.

To illustrate the obtained result let us consider the artificial signal with one minimum in its spectrum obtained using the functions $E_{base}(t)$ (3) and $G_{1}(\nu )$ (4) with the following parameters: k=1, $B_{1}=0.8$, $\nu_{1}$=0.9, Δν=1.0 in the time interval t = [40, 60] which length is equal to $L_{t}$ = 20 units (Example 4). Its Fourier spectrum is depicted in Table A1. The corresponding spectral amplitude modulus $|P(\nu ){|}_{L_{t}=20}$ at the frequencies *ν* = 0.7, 0.9, 1.05 are shown in Table A1 in the second line. The integral spectral amplitude $\left|P_{dyn}(\nu )\right|$ at the chosen frequency ν computed using Equation (A11) for window length T = 2.8 and shift Δ = 0.2 and $h_{t}$ = 0.05; 0.01 is shown in Table A1. In both cases, the computational error in calculating the values of $\left|P_{dyn}(\nu )\right|$ is about $O(h_{t}^{2})$. We see good agreement between both values of the spectral amplitudes.

**Table A1:** Integral spectral amplitude for the artificial signal (Example 4).

			ν	**0.7**	**0.9**	**1.05**
		$h_{t}$				
	$|P(\nu ){|}_{L_{t}=20}$	**0.05**		3.975·10^−2^	4.319·10^−3^	2.006·10^−2^
	$\left|P_{dyn}(\nu )\right|$	**0.05**		3.989·10^−2^	4.276·10^−3^	2.000·10^−2^
		**0.01**		3.981·10^−2^	4.284·10^−3^	2.003·10^−2^

## Appendix C. Influence of Window Shift on the Accuracy of Constructing Spectral Line Dynamics

Using as example the 2,4-DNT substance, we illustrate below an influence of the window shift Δ on the accuracy of constructing spectral line dynamics at frequencies *ν* = 1.1, 4.95, 8.8 and 9.9 THz because it is very important question for the problem under consideration. In Figure A2 the 2,4-DNTspectral line dynamics at the frequency *ν* = 1.1 THz computed at the window length T = 2.8 ps and with increasing window shift Δ = 0.005, 0.05, 0.1, 0.2 and 0.4 ps in the shortened time interval t = [5, 17.5] ps are shown.

The shapes of all the dynamics (a–e) are very similar. However, in (a–c) they contain the oscillations at the frequency *ν* = 5 THz (close to *ν* = 4.95 THz) due to the small shift of window Δ ≤ 0.2 ps. If the window shift increases up to value Δ = 0.2, 0.4 ps, then these oscillations disappear. This fact is illustrated by the Figure A3(a), where the magnified view of the spectral line dynamics computed at the frequency *ν* = 1.1 THz at the increasing window shift Δ = 0.1, 0.2, 0.4 ps is depicted. Let us note that the spectral amplitude corresponding to the frequency *ν* = 5 THz decreases by 10 times at increasing window shift from Δ = 0.05 ps to 0.4 ps. For illustration of this, the corresponding part of the spectra is shown in Figure A3(c),(d) for the frequency range *ν* = [4.8, 5.2] THz and Δ = 0.05 ps and 0.4 ps.

At the same time, if the window shift is decreased up to Δ = 0.05, 0.005 ps, then one can observe the small oscillations occurring at the higher frequencies in the spectral line dynamics. The magnified view of them is depicted in Figure A3 (b). In order to estimate the frequency values of these oscillations, we apply the Fourier transform to the evolution of spectral amplitudes modulus $\left|P_{\nu }(t_{j})\right|$ (they are computed using Equations (A1) and (A2)). The spectrum of the corresponding spectral line dynamics computed at Δ = 0.005 ps is shown in Figure A3 (e) in the frequency range *ν* = [8.7, 10.1] THz and it contains a number of spectral maxima corresponding to small oscillations of the time-dependent spectral intensity. Moreover, one can see the spectral maxima at the frequencies *ν* = 8.82 THz, 9.87 THz, which are close to 2,4-DNT absorption frequencies: *ν* = 8.8 THz and 9.9 THz. It should be stressed that the spectral resolution in (c–e) is equal to Δ*ν* = 0.01 THz.

The similar result occurs for the frequency *ν* = 4.95 THz, which spectral line dynamics are depicted in Figure A4 computed at the window shift Δ = 0.005 ps (a) and 0.4 ps (b). The magnified view of them is also depicted in Figure A4 (a), (b). In (a) it contains the small oscillations corresponding to the higher frequencies. However, for the window shift Δ = 0.4 ps (b) we see much fewer oscillations.

In the spectra of the spectral line dynamics computed at the window shift Δ = 0.005 ps (c–e) one can see the spectral maxima at the frequencies *ν* = 1.05, 4.97, 8.85, 9.87 THz, which are close to 2.4-DNT absorption frequencies *ν* = 1.1, 4.95, 8.8, 9.9 THz. The spectral resolution in (c–h) is equal to Δ*ν* = 0.01 THz. The same result takes place for the window shift Δ = 0.05 ps (not shown).

Further, the spectrum of the spectral line dynamics computed at the window shift Δ = 0.4 ps (j–l) also contains the spectral maxima at the frequencies *ν* = 1.08, 4.92, 8.82, 9.92 THz, which are close to 2.4-DNT absorption frequencies *ν* = 1.1, 4.95, 8.8, 9.9 THz. However, their amplitudes are several orders smaller than in (d–f) and (g–i). Therefore, using of the window shift Δ = 0.4 ps > 0.05 ps does not allows us to see the response of a medium at the frequencies appeared due to the frequency conversion process, i.e., the spurious absorption frequency.

Thus, to see the spectral line dynamic at the high frequencies (for, example *ν* = 4.95, 8.8, 9.9 THz) it is necessary to decrease the window shift if the window length is constant.

## References

[1] Federici J.F., Schulkin B., Huang F., Gary D., Barat R., Oliveira F., Zimdars D. (2005). THz imaging and sensing for security applications—Explosives, weapons and drugs. Semicond. Sci. Technol..

[2] Leahy-Hoppa M.R., Fitch M.J., Zheng X., Hayden L.M., Osiander R. (2007). Wideband terahertz spectroscopy of explosives. Chem. Phys. Lett..

[3] Van Rheenen A.D., Haakestad M.W. (2011). Detection and identification of explosives hidden under barrier materials—What are the THz-technology challenges?. Proc. SPIE.

[4] Leahy-Hoppa M.R., Fitch M.J., Osiander R. (2009). Terahertz spectroscopy techniques for explosives detection. Anal. Bioanal. Chem..

[5] Chen J., Chen Y., Zhao H., Bastiaans G.J., Zhang X.-C. (2007). Absorption coefficients of selected explosives and related compounds in the range of 0.1-2.8 THz. Opt. Express.

[6] Liu H.B., Zhong H., Karpowicz N., Chen Y., Zhang X.C. (2007). Terahertz spectroscopy and imaging for defense and security applications. Proc. IEEE.

[7] Choi K., Hong T., Sim K.I., Ha T., Park B.C., Chung J.H., Cho S.G., Kim J.H. (2014). Reflection terahertz time-domain spectroscopy of RDX and HMX explosives. J. Appl. Phys..

[8] Dean P., Shaukat M.U., Khanna S.P., Chakraborty S., Lachab M., Burnett A., Davies G., Linfield E.H. (2008). Absorption-sensitive diffuse reflection imaging of concealed powders using a terahertz quantum cascade laser. Opt. Express.

[9] Ergün S., Sönmez S. (2015). Terahertz technology for military applications. J. Mil. Inf. Sci..

[10] Kato M., Tripathi S.R., Murate K., Imayama K., Kawase K. (2016). Non-destructive drug inspection in covering materials using a terahertz spectral imaging system with injection-seeded terahertz parametric generation and detection. Opt. Express.

[11] Katz G., Zybin S., Goddard W.A., Zeiri Y., Kosloff R. (2014). Direct MD simulations of terahertz absorption and 2D spectroscopy applied to explosive crystals. Phys. Chem. Lett..

[12] Tanno T., Umeno K., Ide E., Katsumata I., Fujiwara K., Ogawa N. (2014). Terahertz spectra of 1-cyanoadamantane in the orientationally ordered and disordered phases. Philos. Mag. Lett..

[13] McIntosh A.I., Yang B., Goldup S.M., Watkinson M., Donnan R.S. (2012). Terahertz spectroscopy: A powerful new tool for the chemical sciences?. Chem. Soc. Rev..

[14] Nickel D.V., Ruggiero M.T., Korter T.M., Mittleman D.M. (2015). Terahertz disorder-localized rotational modes and lattice vibrational modes in the orientationally-disordered and ordered phases of camphor. Phys. Chem. Chem. Phys..

[15] Kawase K., Shibuya T., Hayashi S.I., Suizu K. (2010). THz imaging techniques for nondestructive inspections. Comptes Rendus Phys..

[16] Ahi K., Anwar M. (2016). Advanced terahertz techniques for quality control and counterfeit detection. Proc. SPIE.

[17] Ahi K., Shahbazmohamadi S., Asadizanjani N. (2018). Quality control and authentication of packaged integrated circuits using enhanced-spatial-resolution terahertz time-domain spectroscopy and imaging. Opt. Lasers Eng..

[18] Stantchev R.I., Sun B., Hornett S.M., Hobson P.A., Gibson G.M., Padgett M.J., Hendry E. (2016). Noninvasive, near-field terahertz imaging of hidden objects using a single-pixel detector. Sci. Adv..

[19] Dong J., Locquet A., Citrin D.S. (2017). Depth resolution enhancement of terahertz deconvolution by autoregressive spectral extrapolation. Opt. Lett..

[20] Su K., Shen Y.C., Zeitler J.A. (2014). Terahertz sensor for non-contact thickness and quality measurement of automobile paints of varying complexity. IEEE Trans. Terahertz Sci. Technol..

[21] Picollo M., Fukunaga K., Labaune J. (2015). Obtaining noninvasive stratigraphic details of panel paintings using terahertz time domain spectroscopy imaging system. J. Cult. Herit..

[22] Zhang H., Sfarra S., Saluja K., Peeters J., Fleuret J., Duan Y., Fernandes H., Avdelidis N., Ibarra-Castanedo C., Maldague X. (2017). Non-destructive investigation of paintings on canvas by continuous wave terahertz imaging and flash thermography. J. Nondestruct. Eval..

[23] Shen Y.C. (2011). Terahertz pulsed spectroscopy and imaging for pharmaceutical applications: A review. Int. J. Pharm..

[24] Puc U., Abina A., Jeglič A., Zidanšek A., Kašalynas I., Venckevičius R., Valušis G. (2018). Spectroscopic analysis of melatonin in the terahertz frequency range. Sensors.

[25] Duka M.V., Dvoretskaya L.N., Babelkin N.S., Khodzitskii M.K., Chivilikhin S.A.E., Smolyanskaya O.A. (2014). Numerical and experimental studies of mechanisms underlying the effect of pulsed broadband terahertz radiation on nerve cells. Quantum Electr..

[26] Gong A., Qiu Y., Chen X., Zhao Z., Xia L., Shao Y. (2019). Biomedical applications of terahertz technology. Appl. Spectrosc. Rev..

[27] Ok G., Park K., Kim H.J., Chun H.S., Choi S.W. (2014). High-speed terahertz imaging toward food quality inspection. Appl. Opt..

[28] Karaliūnas M., Nasser K.E., Urbanowicz A., Kašalynas I., Bražinskienė D., Asadauskas S., Valušis G. (2018). Non-destructive inspection of food and technical oils by terahertz spectroscopy. Sci. Rep..

[29] Wang C., Zhou R., Huang Y., Xie L., Ying Y. (2019). Terahertz spectroscopic imaging with discriminant analysis for detecting foreign materials among sausages. Food Control.

[30] Ahi K. (2017). Review of GaN-based devices for terahertz operation. Opt. Eng..

[31] Shi W., Ding Y.J., Fernelius N., Vodopyanov K. (2002). Efficient, tunable, and coherent 0.18–5.27-THz source based on GaSe crystal. Opt. Lett..

[32] Zangeneh-Nejad F., Safian R. (2016). Significant enhancement in the efficiency of photoconductive antennas using a hybrid graphene molybdenum disulphide structure. J. Nanophotonics.

[33] Kemp M.C. (2011). Explosives detection by terahertz spectroscopy—A bridge too far?. IEEE Trans. Terahertz Sci. Technol..

[34] Palka N. (2013). Identification of concealed materials, including explosives, by terahertz reflection spectroscopy. Opt. Eng..

[35] Ortolani M., Lee J.S., Schade U., Hübers H.-W. (2008). Surface roughness effects on the terahertz reflectance of pure explosive materials. Appl. Phys. Lett..

[36] Schecklman S., Zurk L.M., Henry S., Kniffin G.P. (2011). Terahertz material detection from diffuse surface scattering. J. Appl. Phys..

[37] Federici J.F. (2012). Review of moisture and liquid detection and mapping using terahertz imaging. J. Infrared Millim. Terahertz Waves.

[38] Kong S.G., Wu D.H. (2008). Signal restoration from atmospheric degradation in terahertz spectroscopy. J. Appl. Phys..

[39] Duvillaret L., Garet F., Coutaz J.L. (2000). Influence of noise on the characterization of materials by terahertz time-domain spectroscopy. JOSA B.

[40] Huang Y., Sun P., Zhang Z., Jin C. (2017). Numerical method based on transfer function for eliminating water vapor noise from terahertz spectra. Appl. Opt..

[41] Qu F., Lin L., He Y., Nie P., Cai C., Dong T., Pan Y., Tang Y., Luo S. (2018). Spectral characterization and molecular dynamics simulation of pesticides based on terahertz time-domain spectra analyses and density functional theory (DFT) calculations. Molecules.

[42] Nitta M., Nakamura R., Kadoya Y. (2019). Measurement and Analysis of Noise Spectra in Terahertz Wave Detection Utilizing Low-Temperature-Grown GaAs Photoconductive Antenna. J. Infrared Millim. Terahertz Waves.

[43] Puc U., Abina A., Rutar M., Zidanšek A., Jeglič A., Valušis G. (2015). Terahertz spectroscopic identification of explosive and drug simulants concealed by various hiding techniques. Appl. Opt..

[44] Trofimov V.A., Varentsova S.A. (2015). An effective method for substance detection using the broad spectrum THz signal: A “Terahertz nose”. Sensors.

[45] Trofimov V.A., Varentsova S.A. (2016). Essential limitations of the standard THz TDS method for substance detection and identification and a way of overcoming them. Sensors.

[46] Trofimov V.A., Varentsova S.A. (2016). False detection of dangerous and neutral substances in commonly used materials by means of the standard THz time domain spectroscopy. J. Eur. Opt. Soc..

[47] Trofimov V.A., Varentsova S.A., Trofimov V.V. (2015). Possibility of the detection and identification of substance at long distance using the noisy reflected THz pulse under real conditions. Proc. SPIE.

[48] Trofimov V.A., Varentsova S.A. (2016). Detection and identification of drugs under real conditions by using noisy terahertz broadband pulse. Appl. Opt..

[49] Trofimov V.A., Varentsova S.A. (2018). High effective time-dependent THz spectroscopy method for the detection and identification of substances with inhomogeneous surface. PLoS ONE.

[50] Trofimov V.A., Varentsova S.A. (2019). A possible way for the detection and identification of dangerous substances in ternary mixtures using THz pulsed spectroscopy. Sensors.

[51] Trofimov V.A., Zakharova I.G., Zagursky D.Y., Varentsova S.A. (2017). Detection and identification of substances using noisy THz signal. Proc. SPIE.

[52] Trofimov V.A., Varentsova S.A. (2009). About efficiency of identification of materials using spectrum dynamics of medium response under the action of THz radiation. Proc. SPIE.

[53] Trofimov V.A., Varentsova S.A., Palka N., Szustakowski M., Trzcinski T., Lan S., Liu H. (2011). An influence of the absolute phase of THz pulse on linear and nonlinear medium response. Proc. SPIE.

[54] Trofimov V.A., Varentsova S.A., Szustakowski M., Palka N. (2012). Detection and identification of compound explosive using the SDA method of the reflected THz signal. Proc. SPIE.

[55] Trofimov V., Zagursky D., Zakharova I. Propagation of laser pulse with a few cycle duration in multi-level media. Proceedings of the Days on Diffraction (DD), IEEE.

[56] Trofimov V.A., Zakharova I.G., Zagursky D.Y., Varentsova S.A. (2017). Substance identification by pulsed THz spectroscopy in the presence of disordered structure. Proc. SPIE.

[57] Trofimov V.A., Zakharova I.G., Zagursky D.Y., Varentsova S.A. (2017). New approach for detection and identification of substances using THz TDS. Proc. SPIE.

[58] Trofimov V.A., Varentsova S.A., Zakharova I.G., Zagursky D.Y. (2017). New possibilities of substance identification based on THz TDS using cascade mechanism of high energy level excitation. Sensors.

[59] Marskar R., Osterberg U. (2011). Multilevel Maxwell-Bloch simulations in inhomogeneously broadened media. Opt. Express.

[60] Sun J.H., Shen J.L., Liang L.S., Xu X.Y., Liu H.B., Zhang C.L. (2005). Experimental investigation on terahertz spectra of amphetamine type stimulants. Chin. Phys. Lett..

[61] Newport Corporation Spectra-Physics Mai Tai Ti: Sapphire Oscillator. http://www.spectra-physics.com/products/ultrafast-lasers/mai-tai#specs.

[62] Rice A., Jin Y., Ma X.F., Zhang X.-C., Bliss D., Larkin J., Alexander M. (1994). Terahertz optical rectification from <110> zinc-blende crystals. Appl. Phys. Lett..

[63] TeraView Corporation. https://teraview.com/.

[64] Nyquist H. (1928). Certain topics in telegraph transmission theory. Trans. AIEE.

[65] Shannon C.E. (1948). A mathematical theory of communication. Bell Syst. Tech. J..

[66] Kotelnikov V.A. (1933). On the carrying capacity of the “ether” and wire in telecommunications. Material for the First All-Union Conference on Questions of Communication (Russian).

[67] RIKEN THz Database. http://thzdb.org/.

[68] Wu Q., Zhang X.-C. (1997). 7 terahertz broadband GaP electro-optic sensor. Appl. Phys. Lett..

[69] Yan G., Markov A., Chinifooroshan Y., Tripathi S.M., Bock W.J., Skorobogatiy M. (2013). Resonant THz sensor for paper quality monitoring using THz fiber Bragg gratings. Opt. Lett..

[70] Zangeneh-Nejad F., Safian R. (2016). A graphene-based THz ring resonator for label-free sensing. IEEE Sens. J..

[71] Zangeneh-Nejad F., Safian R. (2016). Hybrid graphene–molybdenum disulphide based ring resonator for label-free sensing. Opt. Commun..

